# Investigating the association between physical health comorbidities and disability in individuals with severe mental illness

**DOI:** 10.1192/j.eurpsy.2021.2255

**Published:** 2021-11-29

**Authors:** Luwaiza Mirza, Jayati Das-Munshi, Jaya Chaturvedi, Honghan Wu, Zeljko Kraljevic, Thomas Searle, Shaweena Shaari, Aurelie Mascio, Naoko Skiada, Angus Roberts, Daniel Bean, Robert Stewart, Richard Dobson, Rebecca Bendayan

**Affiliations:** 1 NIHR Biomedical Research Centre at South London and Maudsley NHS Foundation Trust and King’s College London, London, United Kingdom; 2 Department of Psychological Medicine, Institute of Psychiatry, Psychology and Neuroscience, King’s College London, London, United Kingdom; 3 Department of Biostatistics and Health Informatics, Institute of Psychiatry, Psychology and Neuroscience, King’s College London, London, United Kingdom; 4 Health Data Research UK London, University College London, London, United Kingdom; 5 Institute of Health Informatics, University College London, London, United Kingdom

**Keywords:** Multimorbidity, Bipolar Disorder, Schizophrenia, Health of Nations Outcome Scale, Electronic Health Records

## Abstract

**Background:**

Research suggests that an increased risk of physical comorbidities might have a key role in the association between severe mental illness (SMI) and disability. We examined the association between physical multimorbidity and disability in individuals with SMI.

**Methods:**

Data were extracted from the clinical record interactive search system at South London and Maudsley Biomedical Research Centre. Our sample (n = 13,933) consisted of individuals who had received a primary or secondary SMI diagnosis between 2007 and 2018 and had available data for Health of Nations Outcome Scale (HoNOS) as disability measure. Physical comorbidities were defined using Chapters II–XIV of the International Classification of Diagnoses (ICD-10).

**Results:**

More than 60 % of the sample had complex multimorbidity. The most common organ system affected were neurological (34.7%), dermatological (15.4%), and circulatory (14.8%). All specific comorbidities (ICD-10 Chapters) were associated with higher levels of disability, HoNOS total scores. Individuals with musculoskeletal, skin/dermatological, respiratory, endocrine, neurological, hematological, or circulatory disorders were found to be associated with significant difficulties associated with more than five HoNOS domains while others had a lower number of domains affected.

**Conclusions:**

Individuals with SMI and musculoskeletal, skin/dermatological, respiratory, endocrine, neurological, hematological, or circulatory disorders are at higher risk of disability compared to those who do not have those comorbidities. Individuals with SMI and physical comorbidities are at greater risk of reporting difficulties associated with activities of daily living, hallucinations, and cognitive functioning. Therefore, these should be targeted for prevention and intervention programs.

## Introduction

Providing personalized care to the growing number of individuals with multimorbidity (i.e., two or more physical health conditions) is one of the main challenges of our healthcare system [1]. Traditional research on multimorbidity has focused on ageing populations, however, there is an urgent need to include younger populations which are known to have similar probability of having multiple chronic health conditions when they are socially deprived [2–4] and/or are from an ethnic minority groups [5,6]. This has been highlighted as a key task to reduce the mortality gap between individuals with severe mental illnesses (SMI), such as schizophrenia or bipolar disorder, and the general population.

Individuals diagnosed with SMI, which includes schizophrenia-spectrum (SSD) and bipolar disorders (BD), have also been reported to have a greater risk of comorbid physical health conditions than individuals without SMI [7,8]. In fact, this increased risk of chronic physical morbidity (including cardiovascular, respiratory and infectious diseases, diabetes mellitus and hypertension), has been suggested to underlie, at least in part, premature mortality in individuals with SMI [9]. Specifically, patients with SMI have been shown to have standard mortality ratios that are more than two- to threefold greater than the general population, due to all-cause mortality, including suicide [10].

Moreover, disability associated with mental illness contributes significantly to the global burden of disease, with schizophrenia being described as the mental disorder causing the most disability globally [11,12]. Research in normative population has shown that multimorbidity is associated with an increased likelihood of disability [13] and there are studies that suggest that this could be also the case in individuals with SMI diagnoses such as SSD [14]. According to Strassnig et al. [14], the loss of physical capability in individuals with schizophrenia could be linked to their increased cardiometabolic risk which can potentially accelerate the ageing process.

Within this context, it could be hypothesized that multimorbidity drives increased disability in patients with SMI. The main aim of this study is therefore, to examine the association between physical multimorbidity and disability in individuals with SMI, considering relevant socioeconomic determinants. Our specific objectives were to investigate (a) the prevalence of complex multimorbidity in a large representative cohort of individuals with SMI cohort and their association with the exact SMI diagnosis, age at SMI diagnoses, gender, ethnicity and social deprivation; (b) the association of physical multimorbidity with level of disability which was measured using the Health of the Nations Outcome Scale (HoNOS) [15]—a 12-scale clinician-rated measure of disability which has been developed to measure health and social care outcomes in secondary care mental health services for adults between the ages of 18 and 65; and (c) the potential explanatory role of relevant socioeconomic determinants in this association.

## Methods

### Sample

Patient data were extracted from the Clinical Record Interactive Search (CRIS); a case register system that contains de-identified mental healthcare electronic health record data from the South London and Maudsley Trust NHS Foundation Trust (SLaM). The CRIS system has been developed for use within the National Institute of Health Research (NIHR) Maudsley Biomedical Research Centre (BRC) and provides authorized researchers with regulated and secure access to anonymous information from SLaM NHS Foundation Trust. SLaM is one of Europe’s largest provider of secondary mental healthcare, serving a geographic catchment of approximately 1.2 million residents, and providing all aspects of secondary mental healthcare to all age groups. Since 2006, full electronic clinical records have been deployed in SLaM, and data from these are accessible via the CRIS system which allows searching and retrieval of anonymized full records for over 500,000 cases currently represented in the system [16]. SLaM NHS foundation provides the widest range of NHS mental health and addiction services within the UK. There are over 230 services which constitute inpatient wards, outpatient, and community services. Over 5,000 people each year and provided inpatient care per year and over 45,000 patients are treated in the community across Lambeth, Southwark, Lewisham, and Croydon.

Our maximal sample size (*N* = 13,933) included all individuals aged 15 years or older who had received a primary or secondary diagnosis of SMI between 2007 and 2018 (according to the International Classification of Mental and Behavioural Disorders-10; ICD-10). Specifically, component diagnoses included SSD (ICD-10: F20-F29) and BD (ICD-10: F30-F31). Individuals who did not have data available for the total HoNOS score or had diagnoses for both SSD and BD were excluded. Excluded individuals were more likely to be slightly younger at age at first SMI diagnoses recorded in CRIS, White British men and residents in less deprived areas (Supplementary Table S1).

### Variables

#### Disability

Disability was measured using HoNOS; [15]. HoNOS is a clinician rated tool developed to measure health and social functioning of individuals with SMI and it includes 12 subscales: overactive, aggressive, disruptive or agitated behavior; nonaccidental self-injury; problem drinking or drug taking; cognitive problems; physical illness or disability problems; problems associated with hallucinations or delusions; problems associated with depression; other mental and behaviour problems; problems with relationships; problems with activities of daily living; problems with living conditions; and problems with occupation and activities [15]. Scores for each subcategory range from 0 to 4; with 0 defined as no problems of this kind during the period rated and 4 associated with a severe problem in the category, with the highest impact on the individual. Each score was divided into three categories that have been defined as not present (HoNOS subscale score 0), minimal (score 1), or significant (scores 2–4) [17]. Total HoNOS scores of individuals at the first SMI diagnosis recorded in CRIS were used. Higher scores for HoNOS indicate severe impairment in the individual’s mental health and social functioning, which we label in this study as higher levels of disability. We used HoNOS total adjusted score which becomes relevant when one or more subscores have not been recorded by a clinician. To prevent the score becoming deceptively low, an algorithm within the electronic patient journey system recalculates the total, accounting for the missing values, thereby increasing accuracy of the score [18]. This is a standard approach in research using electronic health records extracted from the electronic patient journey system.

Physical health conditions. Data on the physical health conditions were extracted using a natural language processing algorithm, SemEHR [19]. SemEHR is a clinical NLP framework that embeds a baseline model for identifying contextualized mentions of biomedical concepts from clinical documents. The context information asserts whether a mention is present or absent (negation), current or historical, affirmed or hypothetical, related to the patient or others (e.g., family history). This algorithm showed satisfactory performance estimates (F1 = 0.81–0.95) (details can be found in [20]). This data extraction strategy made available relevant data to identify whether an individual had a mention of a disease from a specific organ system associated with the following ICD-10 Chapters [21]: Chapter II: neoplasms; Chapter III: anemia and blood diseases; Chapter IV: endocrine; Chapter VI: nervous system; Chapter VII: eye and adnexal disorders; Chapter IX: circulatory disorders; Chapter X: respiratory disorders; Chapter XI: digestive disorders; Chapter XII: skin disorders, Chapter XIII: musculoskeletal disorders and finally, Chapter XIV: genitourinary disorders. Complex multimorbidity was defined as having two or more organ systems affected besides the SMI diagnoses [22].

#### Covariates

Covariates included age at first recorded SMI diagnosis, sex, ethnicity (British White, Irish White, Black African, Black Caribbean, South Asian [Bangladeshi, Indian, and Pakistan], and Chinese), and neighborhood-level deprivation [2,3]. Neighborhood-level deprivation was assessed using the index of multiple deprivation (IMD) 2010 score of an area in which the individual resides. This area was measured according to LSOA11 (lower layer super output area 2011) [23]. An official measure for the deprivation of LSOA11 areas in England ranked each LSOA from 1 (most deprived) to 32,844 (least deprived.) The deprivation measure was based on seven census-derived indicators. Each LSOA area contained approximately 1,500 residents or 650 households [24]. The multiple deprivation score is divided into five quintiles to ensure consistency with previous work [17]. Hospitalizations defined as number of admissions for each patient were recorded over the study period.

### Statistical procedure

In order to address our first objective to estimate the prevalence of physical multimorbidity and correlates, we performed descriptive analyses and explored associations using chi-squares, *T*-student, and ANOVA tests. Chi-square tests with Bonferroni adjustments for multiple comparisons were conducted when relevant.

To investigate the association of complex multimorbidity with level of disability measured using the HoNOS and its subscales and the potential explanatory role of relevant socio-economic determinants in this association (objective 2 and 3), we performed series of hierarchical multiple linear regressions. We examined models including independent adjustments for sex (model 2), age (model 3), social deprivation (model 4), ethnicity (model 5), SMI (model 7), and hospitalizations (model 9), so fully adjusted models diagnoses with and without SMI (models 6 and 8).

## Results

### Descriptive analyses

As shown in Table 1, 42.1% of our sample was less than 35 years old at the time of their first SMI diagnoses recorded at SLaM. 52.2% were men, 26.2% were Black, Asian and Minority Ethnic (BAME), 68.2% were in the higher levels of social deprivation, 61.5% of the cohort had complex multimorbidity (i.e., SMI and two or more organ systems affected), and 54.4% were hospitalized during the study period (individuals with complex multimorbidity more likely to have been hospitalized compared to those without complex multimorbidity). Significant associations were found between complex comorbidity and age at SMI diagnoses but no significant differences were found for sex, ethnicity, or social deprivation. We found differences by SMI diagnoses; individuals with SSD were more likely to report complex multimorbidity (62.5%) compared to those diagnosed with BD (58.3%). However, we did not find any differences for those with an intellectual disability defined as mild intellectual disability (F7) or developmental disorders (F8).

**Table 1.** Descriptive statistics for maximal sample size (*n* = 13,933), individuals with complex multimorbidity (*n* = 8,569), and without complex multimorbidity (*n* = 5,364).

	Total cohort *n* (%)	No complex multimorbidity *n* (%)	Complex multimorbidity *n* (%)	Chi-square tests
	13,933 (100.0)	5,364 (38.5)	8,569 (61.5)	
Age at diagnosis				
15–24	2,415 (17.3)	916 (37.9)	1,499 (62.1)	*χ*^2^ = 39.95 (6); *p* < 0.001
25–34	3,462 (24.8)	1,265 (36.5)	2,197 (63.5)	
35–44	2,954 (21.2)	1,133 (38.4)	1,821 (61.6)	
45–54	2,137 (15.3)	798 (37.3)	1,339 (62.7)	
55–64	1,113 (7.99)	431 (38.7)	682 (61.3)	
65–74	996 (7.15)	423 (42.5)	573 (57.5)	
75+	856 (6.14)	398 (46.5)	458 (53.5)	
Sex				
Male	7,267 (52.2)	2,769 (38.1)	4,498 (61.9)	*χ*^2^ = 0.98 (1); *p =* 0.322
Female	6,665 (47.8)	2,595 (38.9)	4,070 (61.1)	
Ethnicity				
British White	4,755 (34.1)	1,862 (39.2)	2,893 (60.8)	*χ*^2^ = 5.95 (5); *p =* 0.312
Black African	1,857 (13.3)	681 (36.7)	1,176 (63.3)	
Black Caribbean	1,227 (8.81)	479 (39.0)	748 (61.0)	
South Asian	470 (3.37)	197 (41.9)	273 (58.1)	
Irish White	298 (2.14)	113 (37.9)	185 (62.1)	
Chinese	100 (0.72)	37 (37.0)	63 (63.0)	
Unknown	5,226 (37.5)	1,995 (38.2)	3,231 (61.8)	
IMD				
1 (most deprived)	370 (2.66)	151 (40.8)	219 (59.2)	*χ*^2^ = 2.21 (4); *p =* 0.697
2	866 (6.22)	327 (37.8)	539 (62.2)	
3	2,854 (20.5)	1,124 (39.4)	1,730 (60.6)	
4	6,540 (46.9)	2,516 (38.5)	4,024 (61.5)	
5 (least deprived)	2,961 (21.3)	1126 (38.0)	1835 (62.0)	
Unknown	342 (2.45)	120 (35.1)	222 (64.9)	
SMI diagnosis				
Schizophrenia spectrum disorder	10,554 (75.74)	3,954 (37.5)	6,600 (62.5)	*χ*^2^ = 19.47 (1); *p* < 0.001
Bipolar affective disorder	3,379 (24.25)	1,410 (41.7)	1,969 (58.3)	
Hospitalizations				
Yes	7,585 (54.44)	2,692 (35.5)	4,893 (64.5)	*χ*^2^ = 63.317 (1); *p* < 0.001
No	6,348 (45.56)	2,672 (42.1)	3,676 (57.9)	
Intellectual disabilities				
F7: Mild intellectual disabilities	288 (2.07)	98 (34.0)	190 (66.0)	*χ*^2^ = 2.29 (1); *p =* 0.130
F8: Developmental disorders	264 (1.89)	94 (35.6)	170 (64.4)	*χ*^2^ = 0.830 (1); *p =* 0.362

*Percentages are shown by column for total cohort and by row for subgroups by complex multimorbidity status.*

The IMD scores of the patients have been split into quintiles, where quintile 1 is the most deprived and quintile 5 being the least deprived.

Abbreviations: IMD, index of multiple deprivation; SMI, severe mental illness.

With regard to the organ systems affected, we found that Chapter VI (nervous system disorders) was the most prevalent (*n* = 4830; 34.7%), followed by Chapter XII dermatological disorders (*n* = 2152, 15.4%) and Chapter IX circulatory disorders (*n* = 2059, 14.8%). For those with BD, neurological disorders (31.9%) were most prevalent, followed by respiratory (14.5%) and musculoskeletal/connective tissue (13.6%). For patients with SSD, we found neurological disorders (35.5%) again to be most prevalent, followed by dermatological (16.1%) and circulatory disorders (15.8%). When we explored differences between BD and SSD (Table 2), we found significant differences for Chapters III (hematological), IV (endocrine), VI (neurological), VII (eye and adnexal), IX (circulatory), and XII (dermatological). Individuals with SSD had higher mentions of hematological disorders, endocrine disorders, neurological disorders, eye disorders, circulatory disorders, and dermatological disorders compared to those with BD.

**Table 2.** Descriptive statistics for individuals with at least one condition from the following organ systems (ICD-10 Chapters) in the SMI cohort (*n* = 13,933) and individuals with SSD (*n* = 10,554) and BD (*n* = 3,379).

	Chapter II Neoplastic disorders	Chapter III Haematological disorders	Chapter IV Endocrine disorders	Chapter VI Neurological disorders	Chapter VII Eye and adnexal disorders	Chapter IX Circulatory disorders	Chapter X Respiratory disorders	Chapter XI Digestive disorders	Chapter XII Dermatological disorders	Chapter XIII Musculoskeletal disorders	Chapter XIV Genitourinary disorders
All	715 (5.1)	655 (4.7)	1,874 (13.5)	4,830 (34.7)	1,376 (9.9)	2,059 (14.8)	2,012 (14.4)	1,812 (13.0)	2,152 (15.4)	2,057 (14.8)	633 (4.5)
SSD	533 (5.1)	531 (5.0)	1,510 (14.3)	3,751 (35.5)	1,107 (10.5)	1,670 (15.8)	1,522 (14.4)	1,379 (13.1)	1,694 (16.1)	1,597 (15.1)	457 (4.3)
BD	182 (5.4)	124 (3.7)	364 (10.8)	1,079 (31.9)	269 (8.0)	389 (11.5)	490 (14.5)	433 (12.8)	458 (13.6)	460 (13.6)	176 (5.2)
*p-value*s	*>0.99*	0.01	<0.001	<0.001	<0.001	<0.001	*>0.99*	*>0.99*	<0.001	0.176	0.198

*Percentages are shown by row, except for the first column. p-values are shown for differences between SSD and BD.*

Abbreviations: BD, bipolar disorder; SSD, Schizophrenia spectrum disorder; SMI, severe mental illness.

The mean HoNOS score of disability at time of first SMI diagnoses recorded, for the whole SMI cohort was 10.60 (SD = 6.14) which showed an increasing pattern with age. There were significant differences in total HoNOS scores between patients diagnosed with SSD and BD (Table 3); individuals with SSD had a higher score on average (SSD = 10.95 vs. BD = 9.49). Significant differences were found for all HoNOS subscales. Patients with SSD were more likely to report severe cognitive problems, physical illness, activities of daily living, hallucinations/delusions, relationship problems, occupational problems, and problems with living conditions. On the other hand, individuals with BD were more likely to have severe problems with agitated behaviors, self-injury, depressed mood, drinking problems, and other mental problems.

**Table 3.** HoNOS total score and subscales for SMI cohort and SSD and BD groups at time of first SMI diagnoses.

	SMI cohort	Schizophrenia spectrum disorder	Bipolar disorder	Statistics
*n*	13,933	10,554	3,379	
HoNOS mean (SD)	10.60 (6.14)	10.95(6.18)	9.49 (5.89)	*t*(2) = 15,386,809.00; *p* < 0.001
Hospitalizations	7,585	6,070 (57.51)	1,337 (39.56)	*χ*^2^(1) = 165.36; *p* = <0.001
Agitated behavior				
0	7,314 (52.5)	5,705 (54.1)	1,609 (47.6)	
1	3,208 (23.0)	2,399 (22.7)	809 (23.9)	
2–4	3,408 (24.5)	2,447 (23.2)	961 (28.4)	
Missing	3 (0.0)	3 (0.0)	0 (0.0)	*χ*^2^(2) = 50.73; *p* < 0.001
Self-injury				
0	11,797 (84.7)	9,142 (86.6)	2,655 (78.6)	
1	1,151 (8.3)	755 (7.2)	396 (11.7)	
2–4	972 (7.0)	646 (6.1)	326 (9.6)	
Missing	13 (0.1)	11 (0.1)	2 (0.1)	*χ*^2^(2) = 129.78; *p* < 0.001
Problem drinking				
0	10,310 (74.0)	7,893 (74.8)	2,417 (71.5)	
1	1,354 (9.7)	953 (9.0)	401 (11.9)	
2–4	2,148 (15.4)	1,613 (15.3)	535 (15.8)	
Missing	121 (0.9)	95 (0.9)	26 (0.8)	χ^2^(2) = 25.36; *p* < 0.001
Cognitive problems				
0	8,598 (61.7)	6,226 (59.0)	2,372 (70.2)	
1	2,928 (21.0)	2,324 (22.0)	604 (17.9)	
2–4	2,372 (17.0)	1,971 (18.7)	401 (11.9)	
Missing	35 (0.3)	33 (0.3)	2 (0.1)	*χ*^2^(2) = 142.49; *p* < 0.001
Physical illness				
0	8,942 (64.2)	6,684 (63.3)	2,258 (66.8)	
1	2,105 (15.1)	1,654 (15.7)	451 (13.3)	
2–4	2,850 (20.5)	2,189 (20.7)	661 (19.6)	
Missing	36 (0.3)	27 (0.3)	9 (0.3)	*χ*^2^(2) = 15.76; *p* < 0.001
Hallucinations				
0	4,845 (34.8)	2,534 (24.0)	2,311 (68.4)	
1	2,176 (15.6)	1,776 (16.8)	400 (11.8)	
2–4	6,865 (49.3)	6,202 (58.8)	663 (19.6)	
Missing	47 (0.3)	42 (0.4)	5 (0.1)	*χ*^2^(2) = 2,283.71; *p* < 0.001
Depressed mood				
0	5,646 (40.5)	4,486 (42.5)	1,160 (34.3)	
1	4,001 (28.7)	3,260 (30.9)	741 (21.9)	
2–4	4,266 (30.6)	2,792 (26.5)	1,474 (43.6)	
Missing	20 (0.1)	16 (0.2)	4 (0.1)	*χ*^2^(2) = 360.09; *p* < 0.001
Other mental problems				
0	3,766 (27.0)	3,011 (28.5)	755 (22.3)	
1	2,819 (20.2)	2,185 (20.7)	634 (18.8)	
2–4	7,281 (52.3)	5,311 (50.3)	1,970 (58.3)	
Missing	67 (0.5)	47 (0.4)	20 (0.6)	*χ*^2^(2) = 72.23; *p* < 0.001
Relationship problems				
0	5,339 (38.3)	3,904 (37.0)	1,435 (42.5)	
1	3,631 (26.1)	2,733 (25.9)	898 (26.6)	
2–4	4,875 (35.0)	3,844 (36.4)	1,031 (30.5)	
Missing	88 (0.6)	73 (0.7)	15 (0.4)	*χ*^2^(2) = 45.97; *p* < 0.001
Daily living problems				
0	7,191 (51.6)	5,188 (49.2)	2,003 (59.3)	
1	3,040 (21.8)	2,344 (22.2)	696 (20.6)	
2–4	3,620 (26.0)	2,960 (28.0)	660 (19.5)	
Missing	82 (0.6)	62 (0.6)	20 (0.6)	*χ*^2^(2) = 125.26; *p* < 0.001
Living conditions				
0	8,468 (60.8)	6,086 (57.7)	2,382 (70.5)	
1	2,277 (16.3)	1,812 (17.2)	465 (13.8)	
2–4	2,788 (19.9)	2,346 (22.2)	442 (13.1)	
Missing	400 (2.9)	310 (2.9)	90 (2.7)	*χ*^2^(2) = 194.23; *p <* 0.001
Occupational problems				
0	6,343 (45.5)	4,523 (42.9)	1820 (53.9)	
1	3,123 (22.4)	2,436 (23.1)	687 (20.3)	
2–4	4,134 (29.7)	3,320 (31.5)	814 (24.1)	
Missing	333 (2.4)	275 (2.6)	58 (1.7)	*χ*^2^(2) = 122.783; *p* < 0.001

*T*-tests and chi-squares were used to examine differences between SSD and BD with Bonferroni adjustments for multiple comparisons.

Abbreviations: BD, bipolar disorders; HoNOS, Health of Nations Outcome Scale; SMI, severe mental illness; SD, standard deviation; SSD, schizophrenia-spectrum disorders.

### Association between multimorbidity and disability

When we investigated whether there were differences in the HoNOS subscales between those individuals having complex multimorbidity and those that did not have complex multimorbidity (Table 4), we did not find significant differences for overall HoNOS scores and subscales except for difficulties with hallucinations which seem to be more likely in individuals with complex multimorbidity. We further examined the association between complex multimorbidity and HoNOS total scores using multiple linear regressions and we found that although there was a positive trend it was only significant when adjusting for age (Supplementary Table S2).

**Table 4.** HoNOS total score and subscales for SMI cohort and complex versus not complex multimorbidity for the whole cohort (*N* = 13,933).

	No complex multimorbidity	Complex multimorbidity	Statistics
*n* (%)	5,364 (38.5)	8,569 (61.5)	
HoNOS mean (SD)	10.49 (6.12)	10.67 (6.16)	*t*(2) = 22,617,650.50; *p* = 0.057
Agitated behavior			
0	2,821 (52.6)	4,493 (52.4)	
1	1,241 (23.1)	1,967 (23.0)	
2–4	1,300 (24.2)	2,108 (24.6)	
Missing	2 (0.0)	1 (0.0)	*□*^2^(2) = 0.24; *p* > 0.99
Self-injury			
0	4,561 (85.0)	7,236 (84.4)	
1	445 (8.3)	706 (8.2)	
2–4	353 (6.6)	619 (7.2)	
Missing	5 (0.1)	8 (0.1)	*χ*^2^(2) = 2.10; *p* > 0.99
Problem drinking			
0	3,998 (74.5)	6,312 (73.7)	
1	508 (9.5)	846 (9.9)	
2–4	806 (15.0)	1,342 (15.7)	
Missing	52 (1.0)	69 (0.8)	*χ*^2^(2) = 1.74; *p* > 0.99
Cognitive problems			
0	3,321 (61.9)	5,277 (61.6)	
1	1,108 (20.7)	1,820 (21.2)	
2–4	920 (17.2)	1,452 (16.9)	
Missing	15 (0.3)	20 (0.2)	*χ*^2^(2) = 0.67; *p* > 0.99
Physical illness			
0	3,381 (63.0)	5,561 (64.9)	
1	810 (15.1)	1,295 (15.1)	
2–4	1156 (21.6)	1,694 (19.8)	
Missing	17 (0.3)	19 (0.2)	*χ*^2^(2) = 6.91; *p* = 0.192
Hallucinations			
0	1,964 (36.6)	2,881 (59.5)	
1	804 (15.0)	1,372 (16.0)	
2–4	2575 (48.0)	4,290 (50.1)	
Missing	21 (0.4)	26 (0.3)	*χ*^2^(2) = 13.55; *p* = 0.006
Depressed mood			
0	2,166 (40.4)	3,480 (40.6)	
1	1,553 (29.0)	2,448 (28.6)	
2–4	1,640 (30.6)	2,626 (30.6)	
Missing	5 (0.1)	15 (0.2)	*χ*^2^(2) =0.22; *p >* 0.99
Other mental problems			
0	1,507 (28.1)	2,259 (26.4)	
1	1,034 (19.3)	1,785 (20.8)	
2–4	2,795 (52.1)	4,486 (52.4)	
Missing	28 (0.5)	39 (0.5)	*χ*^2^(2) = 7.64; *p* = 0.132
Relationship problems			
0	2,072 (38.6)	3,267 (38.1)	
1	1,418 (26.4)	2,213 (25.8)	
2–4	1,845 (34.4)	3,030 (35.4)	
Missing	29 (0.5)	59 (0.7)	*χ*^2^(2) = 1.56; *p* > 0.99
Daily living problems			
0	2,831 (52.8)	4,360 (50.9)	
1	1,124 (21.0)	1,916 (22.4)	
2–4	1,378 (25.7)	2,242 (26.2)	
Missing	31 (0.6)	51 (0.6)	*χ*^2^(2) = 5.57; *p* = 0.372
Living conditions			
0	3,269 (60.9)	5,199 (60.8)	
1	887 (16.5)	1,390 (16.2)	
2–4	1,057 (19.7)	1,731 (20.2)	
Missing	151 (2.8)	249 (2.9)	*χ*^2^(2) = 0.64; *p* > 0.99
Occupational problems			
0	2,508 (46.8)	3,835 (44.8)	
1	1,199 (22.4)	1,924 (22.5)	
2–4	1,528 (28.9)	2,586 (30.2)	
Missing	109 (2.0)	224 (2.6)	*χ*^2^(2) = 4.73; *p* = 0.564

*T*-tests and chi-squares were used to examine differences between SSD and BD with Bonferroni adjustments for multiple comparisons.

Abbreviations: BD, bipolar disorders; HoNOS, Health of Nations Outcome Scale; SMI, severe mental illness; SD, standard deviation; SSD, schizophrenia-spectrum disorders.

**Figure 1.**
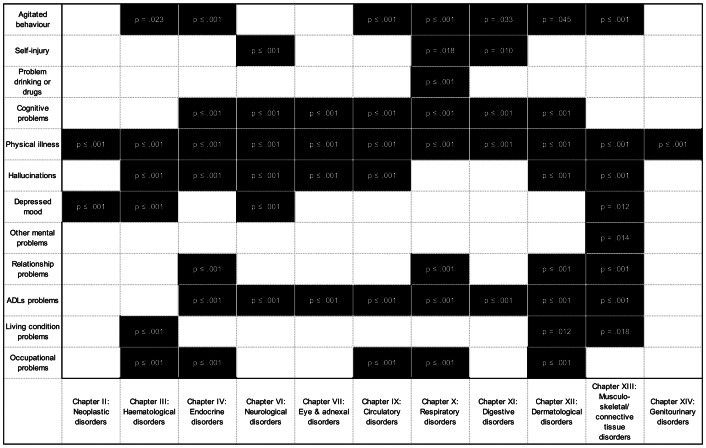
Associations between HoNOS domains and ICD-10 Chapters.

Furthermore, we explored the associations between each specific organ systems considered in this study and HoNOS, total score and its subscales (Supplementary Tables S3 and S4). Summarized results are shown in Figure 1 for significant associations between HoNOS subscales and specific organ systems in models adjusted for sex and age are shown in black. All associations which were not significant have not been shaded. (Supplementary Table S4).

We found that having at least one disorder from some specific organ systems is associated with higher probabilities of reporting difficulties with the disability dimensions captured by HoNOS subscales (See Supplementary Table S4 and Figure S1). The organ systems that showed higher number of HoNOS domains affected are in decreasing order: nine HoNOS domains in individuals with musculoskeletal disorders (Chapter XIII); eight in those with skin/dermatological (Chapter XII); seven domains in those with comorbid endocrine (Chapter IV) or respiratory disorders (Chapter X); six for those with comorbid hematological (Chapter III), neurological (Chapters VI), or circulatory disorders (Chapter IX); five for individuals with comorbid digestive disorders (Chapter XI); four for those with eye and adnexal disorders (Chapters VI and VII); two and one domain for those individuals with comorbid neoplasms (Chapter II) and genito-urinary disorders (Chapter XIV), respectively.

Individuals with comorbid musculoskeletal disorders (Chapter XIII), compared to those without these musculoskeletal disorders, are more likely to report difficulties with agitated behavior, cognitive function, physical illnesses, hallucinations, depressed mood, other mental health problems, relationship problems, Activities of Daily Living (ADLs), and living problems (unadjusted models). All these associations were found to be strengthened after taking in consideration age and sex potential confounding except for cognitive problems depressed mood which were partially attenuated after adjustments. Individuals with comorbid skin/dermatological disorders (Chapter XII), compared to those without these specific comorbid disorders, are more likely to report difficulties with agitated behaviors, cognitive problems, physical illnesses, hallucinations, relationship problems, ADLs, living conditions and occupational problems (unadjusted models). All these associations were found to be strengthened after taking in consideration age and sex potential confounding except for problems with hallucinations which were fully attenuated after adjusting for age and sex.

Individuals with comorbid endocrine disorders (Chapter IV), compared to those without comorbid endocrine diseases, are more likely to report difficulties with agitated behaviors, cognitive problems, physical illnesses, hallucinations, relationship problems, ADLs, and occupational problems (unadjusted models). All these associations were found to be strengthened after taking in consideration age and sex potential confounding. Individuals with comorbid respiratory diseases (Chapter X) compared to those without comorbid respiratory diseases are more likely to report difficulties with agitated behavior, self-injury, drinking problems, cognitive problems, physical illnesses, relationship problems, ADL, and occupational problems (unadjusted models). These associations were strengthened for difficulties associated with agitated behaviors, cognitive problems, physical illness, relationship problems, ADLs, and occupational problems, and partially attenuated for drinking problems and nearly fully attenuated for difficulties associated with self-injury when age and sex adjustments were considered.

Individuals with comorbid neurological diseases (Chapter VI), compared to those without neurological diseases, are more likely to report difficulties with self-injury, cognitive problems, physical illnesses, hallucinations, depressed mood and ADLs (unadjusted models). All these associations (except self-injury) were partially attenuated after adjusting for age and sex. Individuals with comorbid circulatory diseases (Chapter IX), compared to those without circulatory diseases, are more likely to report difficulties with agitated behavior, drinking problems, self-injury, cognitive problems, physical illnesses, hallucinations, depressed mood, ADLs, and occupational problems (unadjusted models). All these associations (except occupational problems) were partially attenuated after adjusting for age and sex.

Overall, most individuals who have at least one condition from the organ systems considered in this study report difficulties with ADLs, hallucinations and cognitive problems and these cannot be fully explained by the normative ageing process. Most individuals with diseases from the specific organ systems considered in this study showed also a consistent higher probability of difficulties associated with physical illness which provides evidence supporting the adequate performance of our data extraction strategy.

### Ad hoc analyses

We performed analyses to consider those individuals that had both SSD and BD diagnoses (Supplementary Table S5) and we found that they had similar demographic characteristics that our study cohort. Individuals with both SMI diagnoses (Mean HoNOS = 10.22, SD = 6.39) reported lower levels of disability than those with only SSD (Mean = 10.95, SD = 6.18; *t*(11448) = 167.13; *p <* 0.001) but greater than those with only BD (Mean = 9.49, SD = 5.89; *t*(11539) = 148.89; *p <* 0.001). Moreover, we also performed analyses focusing only on the SSD group (*n* = 10,554) which showed significant differences in HoNOS score between individuals with and without complex multimorbidity so as for specific ICD-chapters (Supplementary Table S6).

## Discussion

This study aimed to investigate the association between recorded physical multimorbidity and disability in individuals with SMI, considering relevant socio-economic correlates. Our first objective was to estimate the prevalence of physical multimorbidity in a large representative cohort of individuals with SMI and their association with age at SMI diagnosis, nature of SMI diagnosis, gender, ethnicity, and social deprivation. Our results showed that 61.5% of the cohort had complex multimorbidity, which in the context of this study is a SMI diagnosis with comorbidities of two or more organ systems. With regards to the specific organ systems affected in the whole SMI cohort and the SSD subgroup, we found that the systems most commonly affected were those that could be categorized within the ICD-10 Chapter VI—nervous system disorders (34.7%), Chapter XII—dermatological disorders (15.4%), and Chapter IX—circulatory disorders (14.8%). These results for the whole SMI cohort and the SSD subgroup are in line with previous research in which has found nervous system disorders very highly prevalent in these patients [8,17] as well as cardiovascular comorbidity [24]. For the BD subgroup, neurological disorders were also the most common (31.9%) but respiratory disorders (14.5%) were second most common instead followed by musculoskeletal disorders (13.6%). These findings are also partially consistent with previous research which has found Chronic Obstructive Pulmonary Disease (COPD) a common comorbidity in population diagnosed with psychotic disorders [25]. However, other authors have found COPD more prevalent in SSD compared to BD [26]. Future research exploring potential differences associated with respiratory disease comorbidities in SSD and BD are still needed. With regard to the high prevalence of musculoskeletal/connective tissue disorders in the BD subgroup, previous research has found lower bone mineral density and greater prevalence of osteoporosis in individuals with SMI diagnoses including BD. This link has been associated with risk factors such as patients’ lifestyle like smoking, alcohol abuse, vitamin D and calcium deficiency alongside the use of antipsychotics [27] and further research in this direction would be of interest.

When we explored the disability measured with HoNOS and its subscales, there were significant differences in HoNOS scores between patients diagnosed with SSD and BD. Our findings showed a greater prevalence of depressive symptoms and other mental health issues within the BD subgroup. Similar findings have also been found in previous research with BD patients which show high prevalence of depression in primary care settings [28], positive correlation between depressive symptoms and the number of organ systems affected [29] and specifically, depressive symptoms have been also found to be associated with greater levels of disability in individuals with chronic health conditions [30]. Research comparing these associations in individuals with BD and SSD are still scarce and therefore our findings with this respect are not directly comparable with previous research.

When we investigated the association between complex multimorbidity and disability the results were not as clear as when we explored the independent association of physical conditions and HoNOS scores and subscales. Although we found a positive trend in the association between complex multimorbidity and disability, it was only significant when adjusting for age. These findings could suggest that when we are considering a general measure of complex multimorbidity in this cohort (more than two physical health comorbidities beyond mental health comorbidities), we might be focusing on the unhealthiest and therefore those with higher levels of disability, which in turn are more likely to be the oldest of the cohort. In addition, HoNOS total score might not be as informative of the functioning levels of individuals with SMI diagnoses compared to the information that can be extracted from its subscales.

With regards to the association between each specific organ system and HoNOS subscales which represent relevant disability domains, our results indicated that there was a greater variability among organ systems affected which provides evidence to support using specific HoNOS domains rather total composite scores. Specifically, organ systems reflecting comorbid respiratory, endocrine, musculoskeletal, skin/dermatological, neurological, or eye and adnexal disorders were found to be associated with significant difficulties associated with more than five HoNOS domains while others had a lower number of domains affected. This finding not only confirm that individuals with SMIs with physical comorbidities are at greater risk of overall disability, as suggested by previous research in non-SMI populations [13] and SMI populations [31,32], but also highlights the relevance of the differential impact of each specific organ system affected. Physical comorbidities associated with musculoskeletal, skin/dermatological, respiratory, endocrine, circulatory, neurological, or hematological systems seem to have a greater impact on functioning levels compared to physical comorbidities categorized as neoplasms, eye, digestive or genito-urinary disorders. Although not directly comparable, our findings are in line with previous research has found that specific conditions that can be categorized as musculoskeletal [13]. Although cardiovascular comorbidities (circulatory diseases) are highly prevalent in this population [27] and individuals with these were found to have higher total HoNOS scores compared to those without these comorbidities in the present study; individuals with circulatory diseases do not have a very high number of HoNOS subdomains affected. Overall, most individuals who have at least one condition from the organ systems considered in this study report difficulties with ADLs, hallucinations and cognitive problems and these cannot be fully explained by the functional decline driven by the normative ageing process.

One of the main strengths of this study was the large and diverse sample of individuals with SMI which allowed us to provide novel and original findings in these traditionally neglected population in multimorbidity research. In addition, we unlocked hidden data on physical health conditions from clinical text to facilitate further our understanding of the physical comorbidities in this population which is transferable to other mental health trusts in the UK and therefore can facilitate and promote future research in the topic using this type of Electronic Health Records (EHRs). Our data source allowed us to have a key indicator of functioning in these patients which is a widely collected measure in these services, HoNOS, which provides us a unique opportunity for future cross-cohort comparisons.

Some limitations should be also acknowledged. Although our data extraction was quite comprehensive, some systems such as ear related disorders were not available given to limitations of the natural language processing algorithm [19,20] and we mainly focused on system level data (ICD-10 Chapters) rather than specific health conditions. Therefore, future studies should consider widening the number of systems considered and developing strategies that allow to extract and identify specific health conditions at more granular level using this type of records. When we examined specific ICD-chapters, we compared those individuals with a diagnosis from a specific ICD-10 chapter to those individuals without diagnoses of that specific ICD-10 chapter. This might provide us limited information between ICD-10 chapters and therefore further research is needed to detangle further the independent impact of each system affected. It should be acknowledged that although HoNOS is considered a good proxy for disability other specific and more objective measures could be also of interest for comparison purposes. Future studies should also consider measures such as walking speed or grip strength which are physical functioning measures known to predict mortality or specific cognitive functioning instruments which were unfortunately unavailable in our study. Furthermore, although we considered hospitalizations as a proxy of severity, we recognize that this data has limited interpretability considering the nature of our data source with this respect and further research is needed to explore the impact of duration and severity of SMI in this population. Finally, both SSD and BD have varying treatments; for example, the first-line management of SSD involves antipsychotic such as aripiprazole, while treatment-resistant schizophrenia involves use of clozapine [33]. Use of antipsychotics can lead to subsequent side-effects such as weight gain, uncontrollable movements such as tics and tremors, seizures and clozapine also comes with a risk of agranulocytosis which reduces patients’ abilities to fight infections [34]. Bipolar disorder is managed by mood stabilizing drugs like lithium as well as antipsychotics [35]. There is a range of first-line treatments for both SSD and BD which can result in a range of side effects and may impact physical health of patients. As we have not controlled for medications being used by patients, this is a limitation of the study. Future studies exploring the impact of antipsychotics on comorbidities of patients with SMI will be invaluable.

To sum up, our findings are useful and relevant to identify individuals with SMI which might be at high risk of disability. Although we found that older individuals with higher number of organ systems affected beyond their mental health conditions (complex multimorbidity) are more likely to have higher levels of disability compared to those with that cannot be considered as having complex multimorbidity (SMI with none or a single organ system affected), our results highlighted the differential impact that each specific organ systems affected has on disability. Moreover, our findings have provided evidence that domain specific measures of disability measures, rather than composite total scores as indicators, can be more informative to understand the association between physical multimorbidity and disability in research focusing on SMI population. We have found that: (a) individuals with complex multimorbidity should be targeted for prevention and intervention programs aimed to reduce disability in this population; (b) individuals with SMI and physical comorbidities that could be categorized as musculoskeletal, skin/dermatological, respiratory, endocrine, neurological, or circulatory disorders are at higher risk of disability compared to individuals with SMI that do not have those physical comorbidities; and (c) individuals with SMI and physical health comorbidities are at greater risk of reporting difficulties associated with ADLs, hallucinations and cognitive problems. Therefore, policies aiming to reduce disability in SMI populations should prioritize those with musculoskeletal, skin/dermatological, respiratory, endocrine, neurological, or circulatory disorders; and prevention and intervention programs should be targeted to reduce difficulties with ADLs, hallucinations and cognitive problems. Although these results cannot be directly compared with previous research as the association between SMI and complex multimorbidity with disability has not been widely investigated; previous research has also suggested a greater level of cognitive impairment in patients with SSD, which might be leading to lower levels of functioning [33]. Future research should further explore the potential mediator role of cognition in this association, with other potential confounders such as obesity, physical activity or smoking.

## Data Availability

Due to the confidential nature of free-text data, we are unable to make patient-level data available. This project was approved by the CRIS Oversight Committee which is responsible for ensuring all research applications comply with ethical and legal guidelines. The CRIS system enables access to anonymised electronic patient records for secondary analysis from SLaM and has full ethical approvals. CRIS was developed with extensive involvement from service users and adheres to strict governance frameworks managed by service users. It has passed a robust ethics approval process acutely attentive to the use of patient data. Specifically, this system was approved as a dataset for secondary data analysis on this basis by Oxfordshire Research Ethics Committee C (08/H06060/71). The data is de-identified and used in a data-secure format and all patients have the choice to opt-out of their anonymized data being used. Approval for data access can only be provided from the CRIS Oversight Committee at SLaM.

## Supplementary material

For supplementary material accompanying this paper visit https://doi.org/10.1192/j.eurpsy.2021.2255. click here to view supplementary material
