# Supplementary material for: Investigating the association between physical health comorbidities and disability in individuals with severe mental illness
Source: Eur Psychiatry. 2021 Nov 29;64(1):e77. doi: 10.1192/j.eurpsy.2021.2255 (PMC8727716; doi:10.1192/j.eurpsy.2021.2255)
Supplement: Supplementary file 1 [file S0924933821022550sup001.docx]

Supplementary Tables

Supplementary Table 1. Descriptive statistics for the maximal sample included from the SMI cohort and excluded individuals.

|  | | Included SMI cohort  n (%) | | Excluded  individuals | | | | | Chi sq. (df),  p-value | | | |
| --- | --- | --- | --- | --- | --- | --- | --- | --- | --- | --- | --- | --- |
| N (total) | | 13933 | | | | 5403 | | | - | | | |
| **Age at diagnosis**  15 – 24  25 – 34  35 – 44  45 – 54  55 – 64  65 – 74  75 + | | 2415 (17.3)  3462 (24.8)  2954 (21.2)  2137 (15.3)  1113 (8.0)  996 (7.1)  856 (6.1) | | | | 1129 (20.9)  1353 (25.0)  1149 (21.3)  909 (16.8)  565 (10.5)  223 (4.1)  75 (1.4) | | | ꭓ (6)= ^2^ 299.07 ; *p*<.001 | | | |
| **Gender** | | | | | | |  |  |  |  |  |  |
| Male | | 7267 (52.2) | | | | 3037 (56.2). | | |  |  |  |  |
| Female  Unknown | | 6665 (47.8)  1 (0.0) | | | | 2364 (43.8)  2 (0.0) | | | ꭓ (1)= ^2^ 25.74 ; *p*<.001 | | | |
| **Ethnicity**  British (A) | | 4755 (34.1) | | | | 2280(42.2) | | | |  |  |  |
| African (N) | | 1857 (13.3) | | | | 434 (8.0) | | | | ꭓ (5)= ^2^ 305.24; *p*<.001 |  |  |
| Caribbean (M) | | | 1227 (8.8) | | | 205(3.8) | | | |  |  |  |
| South Asian *(Bangladeshi, Indian and Pakistani)* | | 470 (3.4) | | | | 177 (3.3) | | | |  |  |  |
| Chinese  Irish | | 100 (0.7)  298 (2.1) | | | | 28(0.5)  81 (1.5) | | | |  |  |  |
| **Index of Multiple Deprivation (Quintiles)** | | | | | | |  |  |  |  |  |  |
| 1 (least deprived)  2  3  4  5 (most deprived)  Missing | | 370 (2.7)  866 (6.2)  2854 (20.5)  6540 (46.9)  2961 (21.3)  342 (2.5) | | | | 443 (8.2)  611 (11.3)  1132 (21.0)  1879 (34.8)  953 (17.6)  385 (7.1) | | | ꭓ (4)= ^2^ 578.38; *p*<.001 | | | |

Note. Chi-square tests were conducted between the age of diagnosis, gender, ethnicity, index of multiple deprivation in individuals who were included in analyses and individuals who were excluded. Percentages are shown by column.

**Supplementary Table 2.** Regression analyses examining the association between complex multimorbidity and disability (HONOS score) at first SMI diagnoses recorded at SLaM for the whole cohort.

|  | B (SE) |  | Confidence Interval 95% | | | P value |
| --- | --- | --- | --- | --- | --- | --- |
| Model 1 (unadjusted) |  |  |  | | |  |
| No Complex Multimorbidity (ref category) |  | | |  |  |  |
| Complex Multimorbidity | 0.183 (0.107) |  | -0.026- 0.393 | | | *p*=.086 |
| Model 2 (sex adjusted) |  |  |  | | |  |
| No Complex Multimorbidity (ref category) |  |  |  | | |  |
| Complex  Multimorbidity | 0.178 (0.107) |  | -0.031 – 0.387 | | | *p*=.096 |
| Model 3 (age adjusted) |  |  |  | | |  |
| No Complex Multimorbidity (ref category) |  |  |  | | |  |
| Complex  Multimorbidity | 0.236 (0.107) |  | 0.027 – 0.445 | | | *p*= .027 |
| Model 4 (IMD adjusted) |  |  |  | | |  |
| No Complex Multimorbidity (ref category) |  |  |  | | |  |
| Complex  Multimorbidity | 0.205 (0.108) |  | -0.006 – 0.416 | | | *p*= .057 |
| Model 5 (ethnicity adjusted) |  |  |  | | |  |
| No Complex Multimorbidity (ref category) |  |  |  | | |  |
| Complex  Multimorbidity | 0.095 (0.107) |  | -0.170 – 0.360 | | | *p*= .482 |
| Model 6 (fully adjusted for sex, age, IMD and ethnicity) |  |  |  | | |  |
| No Complex Multimorbidity (ref category) |  |  |  | | |  |
| Complex  Multimorbidity | 0.226 (0.106) |  | 0.018 – 0.434 | | | *p*= .242 |
| Model 7 (SMI adjusted) |  |  |  | | |  |
| No Complex Multimorbidity (ref category) |  |  |  | | |  |
| Complex  Multimorbidity | 0.135 (0.106) |  | -0.073- 0.344 | | | *p*=.204 |
| Model 8 (model 6 + SMI) |  |  |  | | |  |
| No Complex Multimorbidity (ref category) |  |  |  | | |  |
| Complex  Multimorbidity | 0.112 (0.135) |  | -0153 – 0.376 | | | *p*= .408 |
| Model 9 (Hospitalizations-adjusted) |  |  |  | | |  |
| No Complex Multimorbidity (ref category) |  |  |  | | |  |
| Complex Multimorbidity | 0.163 (0.107) |  | -0.046 – 0.373 | | | *p=.126* |

* *Model 1: Unadjusted; Model 2-5 independently adjusted for named covariate; Model 6: fully adjusted excluding SMI diagnoses. Model 7 independently adjusted for SMI diagnoses. Model 8: fully adjusted including SMI diagnoses; Model 9: adjusted for number of hospitalizations during study period.*

Supplementary Table 3: Independent associations between organ systems affected and HONOS subscales, across both SMI diagnoses.

|  | Chapter II Neoplastic disorders | | Chapter III  Haematological disorders | | Chapter IV  Endocrine Disorders | | Chapter VI  Neurological Disorders | | | Chapter  VII  Eye and adnexal disorders | | |
| --- | --- | --- | --- | --- | --- | --- | --- | --- | --- | --- | --- | --- |
| N (total) |  |  |  |  |  |  |  | |  |  | |  |
| N | No | Yes | No | Yes | No | Yes | No | Yes | | No | Yes | |
|  | 13218 | 715 | 13278 | 655 | 12,059 | 1,874 | 9103 | | 4830 | 12557 | | 1376 |
| HONOS mean (standard deviation) | 11.58 (6.14) | 10.81 (6.10) | 10.56  (6.15) | 11.23 (5.94) | 10.50  (6.14) | 11.21 (6.09) | 10.45 (6.15) | | 10.88 (6.11) | 10.53 (6.14) | | 11.16  (6.09) |
| T-test | *t*(2)= 4603937.50 ;  *p* =.123 | | *t(*2)= 4038941.50;  *p*<.001 | | *t*(2) = 10497055 *p*<.001 | | *t*(2) = 21086723.50 *p*<.001 | | | *t(*2) = 8119507.50; *p*<.001 | | |
| Agitated Behaviour |  |  |  |  |  |  |  | |  |  | |  |
| 0 | 6950  (52.6) | 364 (50.9) | 6988  (52.6) | 326 (49.8) | 6381 (52.9) | 933 (49.8) | 4824 (53.0) | | 2490 (51.6) | 6602 (52.6) | | 712  (51.7) |
| 1 | 3043 (23.0) | 165 (23.1) | 3065  (23.1) | 143 (21.8) | 2749 (22.8) | 459 (24.5) | 2068 (22.7) | | 1140 (23.6) | 2888 (23.0) | | 320  (23.3) |
| 2 to 4 | 3222  (24.4) | 186 (26.0) | 3222  (24.3) | 186 (28.4) | 2926 (24.3) | 482 (25.7) | 2208 (24.3) | | 1200 (24.8) | 3064 (24.4) | | 344  (25.0) |
| Missing | 47 (0.0) | 0 (0.0) | 3 (0.0) | 0 (0.0) | 3 (0.0) | 0 (0.0) | 3 (0.0) | | 0 (0.0) | 3 (0.0) | | 0 (0.0) |
| Chi-square | ꭓ ^2^(2) 1.11;  *p>.99* | | ꭓ^2^ (2)= 5.75;  *p* = 0.308 | | ꭓ ^2^(2)= 6.46;  *p* = 0.220 | | ꭓ^2^(2)= 2.77;  *p>.99* | | | ꭓ^2^ (2)= 0.38;  *p>.99* | | |
| Self-injury |  |  |  |  |  |  |  | |  |  | |  |
| 0 | 11183 (84.6) | 614 (85.9) | 11227  (84.6) | 570 (87.0) | 10202 (84.6) | 1595 (85.1) | 7737 (85.0) | | 4060 (84.1) | 10638 (84.7) | | 1159  (84.2) |
| 1 | 1088 (8.2) | 63  (8.8) | 1103  (8.3) | 48  (7.3) | 1003 (8.3) | 148  (7.9) | 757  (8.3) | | 394  (8.2) | 1039  (8.3) | | 112  (9.7) |
| 2 to 4 | 934 (7.1) | 38 (5.3) | 935 (7.0) | 37 (5.6) | 842 (7.0) | 130 (6.9) | 601 (6.6) | | 371 (7.7) | 869 (6.9) | | 103 (7.5) |
| Missing | 13 (0.1) | 0 (0.0) | 13 (0.1) | 0 (0.0) | 12 (0.1) | 1 (0.1) | 8 (0.1) | | 5 (0.1) | 11 (0.1) | | 2(0.1) |
|  | ꭓ ^2^ (2)= 3.38; *p>.99* | | ꭓ ^2^ (2)= 2.91; *p>.99* | | ꭓ^2^ (2)= 0.40; *p>.99* | | ꭓ ^2^ (2)= 5.69; *p* = 0.319 | | | ꭓ ^2^ (2)= 0.63; *p>.99* | | |
| Problem drinking |  |  |  |  |  |  |  | |  |  | |  |
| 0 | 9741 (73.7) | 569 (79.6) | 9843 (74.1) | 467 (71.3) | 8922 (74.0) | 1388 (74.1) | 6711 (73.7) | | 3599 (74.5) | 9288 (74.0) | | 1022 (74.3) |
| 1 | 1,303 (9.9) | 51 (7.1) | 1292 (9.7) | 62 (9.5) | 1189 (9.9) | 165 (8.8) | 874 (9.6) | | 480 (9.9) | 1239 (9.9) | | 115 (8.4) |
| 2 to 4 | 2058 (15.6) | 90 (12.6) | 2034 (15.3) | 114 (17.4) | 1841 (15.3) | 307 (16.4) | 1440 (15.8) | | 708 (14.7) | 1926 (15.3) | | 222 (16.1) |
| Missing | 116 (0.9) | 5 (0.7) | 109 (0.8) | 12 (1.8) | 107 (0.9) | 14 (0.7) | 78 (0.9) | | 43 (0.9) | 104 (0.8) | | 17 (1.2) |
|  | ꭓ ^2^ (2)= 12.25; *p*=.011 | | ꭓ ^2^ (2)= 2.44 *p>.99* | | ꭓ ^2^ (2)= 3.16; *p>.99* | | ꭓ ^2^ (2)= 3.39; *p>.99* | | | ꭓ ^2^(2)= 3.42; *p*=.996 | | |
| Cognitive problems |  |  |  |  |  |  |  | |  |  | |  |
| 0 | 8175 (61.8) | 423 (59.1) | 8206 (61.8) | 392 (59.8) | 7533 (62.5) | 1065 (56.8) | 5733 (63.0) | | 2865 (59.3) | 7829 (62.3) | | 769 (55.9) |
| 1 | 2763 (20.9) | 165 (23.1) | 2798 (21.1) | 130 (19.8) | 2523 (20.9) | 405 (21.6) | 1863 (20.5) | | 1065 (22.0) | 2598 (20.7) | | 330 (24.0) |
| 2-4 | 2246 (17.0) | 126 (17.6) | 2241 (16.9) | 131 (20.0) | 1972 (16.4) | 400 (21.3) | 1484 (16.3) | | 888 (18.4) | 2097 (16.7) | | 275 (20.0) |
| Missing | 34 (0.3) | 1 (0.1) | 33 (0.2) | 2 (0.3) | 31 (0.3) | 4 (0.2) | 23 (0.3) | | 12 (0.2) | 33 (0.3) | | 2 (0.1) |
|  | ꭓ ^2^ (2)= 2.47; *p>.99* | | ꭓ ^2^ (2)= 4.41; *p*=.605 | | ꭓ ^2^ (2)= 32.45; *p*<.001 | | ꭓ ^2^ (2)= 18.67; *p*<.001 | | | ꭓ ^2^ (2)= 22.63; *p*<.001 | | |
| Physical illness |  |  |  |  |  |  |  | |  |  | |  |
| 0 | 8576 (64.9) | 366 (51.2) | 8553 (64.3) | 409 (62.4) | 7842 (65.0) | 1100 (58.7) | 6046 (66.4) | | 2896 (60.0) | 8152 (64.9) | | 790 (57.4) |
| 1 | 1985 (15.0) | 120 (16.8) | 2020 (15.2) | 85 (13.0) | 1770 (14.7) | 335 (17.9) | 1298 (14.3) | | 807 (16.7) | 1888 (15.0) | | 217 (15.8) |
| 2 to 4 | 2623 (19.8) | 227 (31.7) | 2691 (20.3) | 159 (24.3) | 2414 (20.0) | 436 ( 23.3) | 1734 (19.0) | | 1116 (23.1) | 2487 (19.8) | | 363 (26.4) |
| Missing | 34 (0.3) | 2 (0.3) | 34 (0.3) | 2 (0.3) | 33 (0.3) | 3 (0.2) | 25 (0.3) | | 11 (0.2) | 30 (0.2) | | 6 (0.4) |
|  | ꭓ ^2^(2)= 68.22 ;*p*<.001 | | ꭓ ^2^(2)= 7.29; *p* =.143 | | ꭓ ^2^(2)=29.45; *p*<.001 | | ꭓ ^2^(2)= 58.42; *p*<.001 | | | ꭓ ^2^(2)= 37.60; *p*<.001 | | |
| Hallucinations |  |  |  |  |  |  |  | |  |  | |  |
| 0 | 4600 (34.8) | 245 (34.3) | 4669 (35.2) | 176 (26.9) | 4255 (35.3) | 590 (31.5) | 3254 (35.7) | | 1591 (32.8) | 4444 (35.4) | | 401 (29.1) |
| 1 | 2065 (15.6) | 111 (15.5) | 2053 (15.5) | 123 (18.8) | 1872 (15.5) | 304 (16.2) | 1403 (15.4) | | 773 (16.0) | 1930 (15.4) | | 246 (17.9) |
| 2 to 4 | 6507 (49.2) | 358 (50.1) | 6510 (49.0) | 355 (54.2) | 5892 (48.9) | 973 (51.9) | 4412 (48.5) | | 2453 (50.8) | 6137 (48.9) | | 728 (52.9) |
| Missing | 46 (0.3) | 1 (0.1) | 46 (0.3) | 1 (0.2) | 40 (0.3) | 7 (0.4) | 34 (0.4) | | 13 (0.3) | 46 (0.4) | | 1 (0.1) |
|  | ꭓ ^2^ (2)= 0.15; *p>.99* | | ꭓ ^2^(2)= 20.09;*p*<.001 | | ꭓ ^2^(2)= 10.33 ; *p*=.033 | | ꭓ ^2^(2)=11.29; *p*=.022 | | | ꭓ^2^(2)= 22.95 ;*p*<.001 | | |
| Depressed mood |  |  |  |  |  |  |  | |  |  | |  |
| 0 | 5384 (40.7) | 262 (36.6) | 5380 (40.5) | 266 (40.5) | 4899 (40.6) | 747 (39.9) | 3737 (41.1) | | 1909 (33.8) | 5103 (40.6) | | 543 (39.5) |
| 1 | 3763 (28.5) | 238 (33.3) | 3779 (28.5) | 222 (33.9) | 3413 (28.3) | 588 (31.4) | 2544 (27.9) | | 1457 (30.2) | 3570 (28.4) | | 431 (31.3) |
| 2 to 4 | 4051 (30.6) | 215 (30.1) | 4099 (30.9) | 167 (25.5) | 3732 (30.9) | 534 (28.5) | 2819 (30.9) | | 1456 (30.1) | 3866 (30.8) | | 400 (29.1) |
| Missing | 20 (0.2) | 0 (0.0) | 20 (0.2) | 0 (0.0) | 15 (0.1) | 5 (0.3) | 12 (0.1) | | 8 (0.2) | 18 (0.1) | | 2 (0.6) |
|  | ꭓ ^2^ (2)= 8.34; *p*=.083 | | ꭓ ^2^ (2)= 12.29; *p*=.011 | | ꭓ ^2^(2)= 8.76; *p*=.066 | | ꭓ ^2^ (2)=7.77; *p*=.116 | | | ꭓ ^2^ (2)= 5.23; *p*=.402 | | |
| Other mental problems |  |  |  |  |  |  |  | |  |  | |  |
| 0 | 3554 (26.9) | 212 (29.7) | 3599 (27.1) | 167 (25.5) | 3279 (27.2) | 487 (26.0) | 2480 (27.2) | | 1286 (26.6) | 3388 (27.0) | | 378 (27.5) |
| 1 | 2680 (20.3) | 139 (19.4) | 2684 (20.2) | 135 (20.6) | 2410 (20.0) | 409 (21.8) | 1833 (20.1) | | 986 (20.4) | 2529 (20.1) | | 290 (21.1) |
| 2 to 4 | 6918 (52.3) | 363 (50.8) | 6930 (52.2) | 351 (53.6) | 6309 (52.3) | 972 (51.9) | 4743 (52.1) | | 2538 (52.5) | 6580 (52.4) | | 701 (50.9) |
| Missing | 66 (0.5) | 1 (0.1) | 65 (0.5) | 2 (0.3) | 61 (0.5) | 6 (0.3) | 47 (0.5) | | 20 (0.4) | 60 (0.5) | | 7 (0.5) |
|  | ꭓ ^2^(2)= 2.45; *p>.99* | | ꭓ ^2^(2)=0.88 ; *p>.99* | | ꭓ ^2^ (2)= 3.64; *p*=.891 | | ꭓ ^2^ (2)= 0.68; *p>.99* | | | ꭓ ^2^ (2)= 1.15; *p>.99* | | |
| Relationship Problems |  |  |  |  |  |  |  | |  |  | |  |
| 0 | 5053 (38.2) | 286 (40.0) | 5,099 (38.4) | 240 (36.6) | 4659 (38.6) | 680 (36.3) | 3470 (38.1) | | 1869 (38.7) | 4847 (38.6) | | 492 (35.8) |
| 1 | 3443 (26.0) | 188 (26.3) | 3,458 (26.0) | 173 (26.4) | 3166 (26.3) | 465 (24.8) | 2396 (26.3) | | 1235 (25.6) | 3259 (26.0) | | 372 (27.0) |
| 2 to 4 | 4639 (35.1) | 236 (33.0) | 4637 (34.9) | 238 (36.3) | 4159 (34.5) | 716 (38.2) | 3177 (34.9) | | 1698 (34.8) | 4373 (34.8) | | 502 (36.5) |
| Missing | 83 (0.6) | 5 (0.7) | 84 (0.6) | 4 (0.6) | 75 (0.6) | 13 (0.7) | 60 (0.7) | | 28 (0.6) | 78 (0.6) | | 10 (0.7) |
|  | ꭓ ^2^ (2)= 1.41; *p>.99* | | ꭓ ^2^ (2)= 0.89; *p>.99* | | ꭓ ^2^ (2)= 10.04; *p*=.039 | | ꭓ ^2^ (2)=1.014; *p>.99* | | | ꭓ ^2^(2)= 4.15; *p*=.693 | | |
| Daily living problems |  |  |  |  |  |  |  | |  |  | |  |
| 0 | 6852 (51.8) | 339 (47.4) | 6875 (51.8) | 316 (48.2) | 6349 (52.6) | 842 (44.9) | 4855 (53.3) | | 2336 (48.4) | 6574 (52.4) | | 617 (44.8) |
| 1 | 2873 (21.7) | 167 (23.4) | 2886 (21.7) | 154 (23.5) | 2597 (21.5) | 443 (23.6) | 1908 (21.0) | | 1132 (23.4) | 2683 (21.4) | | 357 (25.9) |
| 2 to 4 | 3415 (25.8) | 205 (28.7) | 3441 (25.9) | 179 (27.3) | 3042 (25.2) | 578 (30.8) | 2289 (25.1) | | 1331 (27.6) | 3227 (25.7) | | 393 (28.6) |
| Missing | 78 (0.6) | 4 (0.6) | 76 (0.6) | 6 (0.9) | 71 (0.6) | 11 (0.6) | 51 (0.6) | | 31 (0.6) | 73 (0.6) | | 9 (0.7) |
|  | ꭓ ^2^ (2)= 5.49; *p*=.352 | | ꭓ ^2^ (2)= 2.89; *p>.99* | | ꭓ ^2^ (2)= 41.71; *p*<.001 | | ꭓ ^2^ (2)= 31.04; *p*<0.001 | | | ꭓ ^2^ (2)= 29.40; *p*<.001 | | |
| Living condition problems |  |  |  |  |  |  |  | |  |  | |  |
| 0 | 8,022 (60.7) | 446 (62.4) | 8,101 (61.0) | 367 (56.0) | 7,343 (60.9) | 1,125 (60.0) | 5,517 (60.6) | | 2,951 (61.1) | 7,646 (60.9) | | 822 (59.7) |
| 1 | 2,159 (16.3) | 118 (16.5) | 2,173 (16.4) | 104 (15.9) | 1,975 (16.4) | 302 (16.1) | 1,477 (16.2) | | 800 (16.6) | 2,038 (16.2) | | 239 (17.4) |
| 2 to 4 | 2,656 20.1) | 132 (18.5) | 2,629 (19.8) | 159 (24.3) | 2,403 (19.9) | 385 (20.5) | 1,849 (20.3) | | 939 (19.4) | 2,513 (20.0) | | 275 (20.0) |
| Missing | 381 (2.9) | 19 (2.7) | 375 (3.8) | 25 (4.0) | 338 (2.8) | 62 (3.3) | 260 (2.9) | | 140 (2.9) | 360 (2.9) | | 40 (2.9) |
|  | ꭓ ^2^ (2)= 1.23; *p>.99* | | ꭓ ^2^ (2)= 8.91; *p* =.066 | | ꭓ ^2^(2)= 0.53; *p>.99* | | ꭓ ^2^ (2)= 1.54; *p>.99* | | | ꭓ ^2^ (2)= 1.26; *p>.99* | | |
| Occupational problems |  |  |  |  |  |  |  | |  |  | |  |
| 0 | 6001 (45.4) | 342 (47.8) | 6088 (45.9) | 255 (38.9) | 5554 (46.1) | 789 (42.1) | 4244 (46.6) | | 2099 (43.5) | 5748 (45.8) | | 595 (43.2) |
| 1 | 2970 (22.5) | 153 (21.4) | 2961 (22.3) | 162 (24.7) | 2683 (22.2) | 440 (23.5) | 1973 (21.7) | | 1150 (23.8) | 2792 (22.2) | | 331 (24.1) |
| 2 to 4 | 3,930 (29.7) | 204 (28.5) | 3,918 (29.5) | 216 (33.0) | 3,542 (29.4) | 592 (31.6) | 2683 (29.5) | | 1451 (30.0) | 3720 (29.6) | | 414 (30.1.9) |
| Missing | 317 (2.4) | 16 (2.2) | 311 (2.3) | 22 (3.4) | 280 (2.3) | 53 (2.8) | 203 (2.2) | | 130 (2.7) | 297 (2.4) | | 36 (2.6) |
|  | ꭓ ^2^ (2)= 1.55; *p>.99* | | ꭓ ^2^ (2)= 10.78; *p*=.028 | | ꭓ ^2^(2)= 9.35; *p*=.050 | | ꭓ ^2^ (2)= 13.65; *p*=.006 | | | ꭓ ^2^ (2)= 3.67; *p*=.875 | | |

*Percentages are shown by column.

**missing values not included in statistical evaluation

**Supplementary Table 3. (continued) Independent associations between organ systems affected and HONOS subscales, across both SMI diagnoses.**

|  | | | | Chapter IX  Circulatory disorders | | | | | Chapter X  Respiratory  Disorders | | | | Chapter XI  Digestive disorders | | | | Chapter XII  Dermatological disorders | | | | | Chapter XIII  Musculoskeletal/ connective tissue disorders | | | | | Chapter XIV  Genitourinary disorders | | | | | |
| --- | --- | --- | --- | --- | --- | --- | --- | --- | --- | --- | --- | --- | --- | --- | --- | --- | --- | --- | --- | --- | --- | --- | --- | --- | --- | --- | --- | --- | --- | --- | --- | --- |
| N (total) | No | | | | | Yes | No | | | Yes | | No | | | Yes | | | No | | Yes | | | No | | Yes | | | No | | Yes | |  |
| N total | 11,874 | | | | | 2,059 | 11,921 | | | 2012 | | 12,121 | | | 1,812 | | | 11,781 | | 2,152 | | | 11,876 | | 2,057 | | | 13,300 | | 633 | |  |
| HONOS mean (standard deviation) | 10.47 (6.11) | | | | | 11.37 (6.27) | 10.49 (6.12) | | | 11.23 (6.24) | | 10.50 (6.11) | | | 11.28 (6.30) | | | 10.48 (6.11) | | 11.26 (6.26) | | | 10.47 (6.10) | | 11.35 (6.32) | | | 10.58 (6.14) | | 11.02 (6.14) | |  |
| T-test | | | | *t*(2) =11230165.50 ;*p*<.001 | | | | | *t*(2)=  11170659.00; *p*<.001 | | | | *t*(2) = 10207867.00; *p*<.001 | | | | *t*(2) = 11770461.50; *p*<.001 | | | | | *t*(2)= 11252762.50; *p*<.001 | | | | | *t*(2)= 4018583.50; *p*=.0265 | | | | | |
|  |  | | | |  | | |  | | |  | | |  | |  | | |  | |  | | |  | |  | | |  | |  |  |
| Agitated behaviour |  | | | | |  |  | | |  | |  | | |  | | |  | |  | | |  | |  | | |  | |  | |  |
| 0 | 6290 (53.0) | | | | | 1024 (49.7) | 6328 (53.1) | | | 986 (49.0) | | 6413 (52.9) | | | 901 (49.7) | | | 6239 (53.0) | | 1075 (50.0) | | | 6303 (53.1) | | 1011 (49.1) | | | 6989 (52.5) | | 325 (51.3) | |  |
| 1 | 2718 (22.9) | | | | | 490 (23.8) | 2744 (23.0) | | | 464 (23.1) | | 2771 (22.9) | | | 437 (24.1) | | | 2684 (22.8) | | 524 (24.3) | | | 2725 (22.9) | | 483 (23.5) | | | 3,057 (23.0) | | 151 (23.9) | |  |
| 2 to 4 | 2863 (24.1) | | | | | 545 (26.5) | 2846 (23.9) | | | 562 (27.9) | | 2934 (24.2) | | | 474 (26.2) | | | 2855 (24.2) | | 553 (25.7) | | | 2845 (24.0) | | 563 (27.4) | | | 3,251 (24.4) | | 157 (24.8) | |  |
| Missing | 3 (0.0) | | | | | 0 (0.0) | 3 (0.0) | | | 0 (0.0) | | 3 (0.0) | | | 0 (0.0) | | | 3 (0.0) | | 0 (0.0) | | | 3 (0.0) | | 0 (0.0) | | | 3 (0.0) | | 0 (0.0) | |  |
| Chi-square | | | | ꭓ ^2^ (2)= 8.12;  *p*=.094 | | | | | ꭓ ^2^ (2)= 17.04 *p*<.001 | | | | ꭓ ^2^ (2)= 6.58;  *p*=.204 | | | | ꭓ ^2^(2)= 6.66; *p*=.198 | | | | | ꭓ ^2^ (2)= 13.71;  *p*=.006 | | | | | ꭓ ^2^ (2)= 0.40;  *p>.99* | | | | | |
| Self injury |  | | | | |  |  | | |  | |  | | |  | | |  | |  | | |  | |  | | |  | |  | |  |
| 0 | 10042 (84.6) | | | | | 1755 (85.2) | 10148 (85.1) | | | 1649 (82.0) | | 10291 (84.9) | | | 1506 (83.1) | | | 9974 (84.7) | | 1823 (84.7) | | | 10052 (84.6) | | 1,745 (84.8) | | | 11268 (84.7) | | 529 (83.6) | |  |
| 1 | 985 (8.3) | | | | | 166 (8.1) | 955 (8.0) | | | 196 (9.7) | | 992 (8.2) | | | 159 (8.8) | | | 980 (8.3) | | 171 (7.9) | | | 983 (8.3) | | 168 (8.2) | | | 1098 (8.3) | | 53 (8.4) | |  |
| 2 to 4 | 835 (7.0) | | | | | 137 (6.7) | 808 (6.8) | | | 164 (8.2) | | 825 (6.8) | | | 147 (8.1) | | | 817 (6.9) | | 155 (7.2) | | | 828 (7.0) | | 144 (7.0) | | | 923 (6.9) | | 49 (7.7) | |  |
| Missing | 12 (0.1) | | | | | 1 (0.0) | 10 (0.1) | | | 3 (0.1) | | 13 (0.1) | | | 0 (0.0) | | | 10 (0.1) | | 3 (0.1) | | | 13 (0.1) | | 0 (0.0) | | | 11 (0.1) | | 2 (0.3) | |  |
|  | | | | ꭓ ^2^ (2)= 0.57; *p>.99* | | | | | ꭓ ^2^ (2)= 12.94;  *p* =.011 | | | | ꭓ ^2^ (2)= 5.11;  *p* =.429 | | | | ꭓ ^2^ (2)= 0.49;  *p>.99* | | | | | ꭓ ^2^ (2)= 0.03;  *p>.99* | | | | | ꭓ ^2^ (2)= 0.66;  *p>.99* | | | | | |
| Problem drinking |  | | | |  | | |  | | |  | | |  | |  | | |  | |  | | |  | |  | | |  | |  |  |
| 0 | 8736 (73.6) | | | | | 1574 (76.4) | 8890 (74.6) | | | 1420 (70.6) | | 8966 (74.0) | | | 1344 (74.2) | | | 8705 (73.9) | | 1605 (74.6) | | | 8775 (73.9) | | 1535 (74.6) | | | 9810 (73.8) | | 500 (79.0) | |  |
| 1 | 1173 (9.9) | | | | | 181 (8.8) | 1139 (9.6) | | | 215 (10.7) | | 1186 (9.8) | | | 168 (9.3) | | | 1152 (9.8) | | 202 (9.4) | | | 1160 (9.8) | | 194 (9.4) | | | 1311 (9.9) | | 43 (6.8) | |  |
| 2 to 4 | 1862 (15.7) | | | | | 286 (13.9) | 1789 (15.0) | | | 359 (17.8) | | 1866 (15.4) | | | 282 (15.6) | | | 1829 (15.5) | | 319 (14.8) | | | 1841 (15.5) | | 307 (14.9) | | | 2062 (15.5) | | 86 (13.6) | |  |
| Missing | 103 (0.9) | | | | | 18 (0.9) | 103 (0.9) | | | 18 (0.9) | | 103 (0.8) | | | 18 (1.0) | | | 95 (0.8) | | 26 (1.2) | | | 100 (0.8) | | 21 (1.0) | | | 117 (0.9) | | 4 (0.6) | |  |
| Chi square | | | | ꭓ ^2^ (2)= 7.75;  *p* =.116 | | | | | ꭓ ^2^ (2)= 14.97;  *p* =.006 | | | | ꭓ ^2^ (2)= 0.46 (2)  *p>.99* | | | | ꭓ ^2^ (2)= 0.96;  *p>.99* | | | | | ꭓ ^2^ (2)= 0.71;  *p>.99* | | | | | ꭓ ^2^ (2)= 9.49;  *p*=.050 | | | | | |
| Cognitive problems |  | | | |  | | |  | | |  | | |  | |  | | |  | |  | | |  | |  | | |  | |  |  |
| 0 | 7488 (63.1) | | | | | 1110 (53.9) | 7439 (62.4) | | | 1159 (57.6) | | 7545 (62.2) | | | 1053 (58.1) | | | 7372 (62.6) | | 1226 (57.0) | | | 7387 (62.2) | | 1211 (58.9) | | | 8226 (61.8) | | 372 (58.8) | |  |
| 1 | 2418 (20.4) | | | | | 510 (24.8) | 2478 (20.8) | | | 450 (22.4) | | 2528 (20.9) | | | 400 (22.1) | | | 2428 (20.6) | | 500 (23.2) | | | 2457 (20.7) | | 471 (22.9) | | | 2789 (21.0) | | 139 (22.0) | |  |
| 2-4 | 1940 (16.3) | | | | | 432 (21.0) | 1972 (16.5) | | | 400 (19.9) | | 2016 (16.6) | | | 356 (19.6) | | | 1950 (16.6) | | 422 (19.6) | | | 2006 (16.9) | | 366 (17.8) | | | 2251 (16.9) | | 121 (19.1) | |  |
| Missing | 28 (0.2) | | | | | 7 (0.3) | 32 (0.3) | | | 3 (0.1) | | 32 (0.3) | | | 3 (0.2) | | | 31 (0.3) | | 4 (0.2) | | | 26 (0.2) | | 9 (0.4) | | | 34 (0.3) | | 1 (0.2) | |  |
|  | | | | ꭓ ^2^ (2)= 62.29; *p*<.001 | | | | | ꭓ ^2^ (2)= 19.72; *p*<.001 | | | | ꭓ ^2^ (2)= 13.88;  *p* =.006 | | | | ꭓ ^2^ (2)= 25.21; *p*<.001 | | | | | ꭓ ^2^ (2)= 8.06  *p* =.099 | | | | | ꭓ ^2^ (2)= 2.91;  *p>.99* | | | | | |
| Physical illness |  | | | |  | | |  | | |  | | |  | |  | | |  | |  | | |  | |  | | |  | |  |  |
| 0 | 7847 (66.1) | | | | | 1095 (53.2) | 7727 (64.8) | | | 1215 (60.4) | | 7935 (65.5) | | | 1007 (55.6) | | | 7691 (65.3) | | 1251 (58.1) | | | 7810 (65.8) | | 1132 (55.0) | | | 8651 (65.0) | | 291 (46.0) | |  |
| 1 | 1747 (14.7) | | | | | 358 ( 17.4) | 1776 (14.9) | | | 329 (16.4) | | 1799 (14.8) | | | 306 (16.9) | | | 1752 (14.9) | | 353 (16.4) | | | 1763 (14.8) | | 342 (16.6) | | | 2001 (15.0) | | 104 (16.4) | |  |
| 2 to 4 | 2252 (19.0) | | | | | 598 (29.0) | 2386 (20.0) | | | 464 (23.1) | | 2355 (19.4) | | | 495 (27.3) | | | 2308 (19.6) | | 542 (25.2) | | | 2275 (19.2) | | 575 (28.0) | | | 2614 (19.7) | | 236 (37.3) | |  |
| Missing | 28 (0.2) | | | | | 8 (0.4) | 32 (0.3) | | | 4 (0.2) | | 32 (0.3) | | | 4 (0.2) | | | 30 (0.3) | | 6 (0.3) | | | 28 (0.2) | | 8 (0.4) | | | 34 (0.3) | | 2 (0.3) | |  |
| Chi square | | | | ꭓ ^2^ (2)= 141.10 ;*p*<.001 | | | | | ꭓ ^2^ (2)= 15.48 (2); *p* <.001 | | | | ꭓ ^2^ (2)= 76.33 (2) ;*p*<.001 | | | | ꭓ ^2^ (2)= 45.18; *p*<.001 | | | | | ꭓ ^2^ (2)= 101.58; *p*<.001 | | | | | ꭓ ^2^ (2)= 129.89; *p*<.001 | | | | | |
| Hallucinations | | |  | |  | | |  | | |  | | |  | |  | | |  | |  | | |  | |  | | |  | |  |  |
| 0 | 4264 (35.9) | | | | | 581 (28.2) | 4174 (35.0) | | | 671 (33.3) | | 4234 (34.9) | | | 611 (33.7) | | | 4180 (35.5) | | 665 (30.9) | | | 4196 (35.3) | | 649 (31.6) | | | 4612 (34.7) | | 233 (36.8) | |  |
| 1 | 1850 (15.6) | | | | | 326 (15.8) | 1844 (15.5) | | | 332 (16.5) | | 1896 (15.6) | | | 280 (15.5) | | | 1,802 (15.3) | | 374 (17.4) | | | 1838 (15.5) | | 338 (16.4) | | | 2084 (15.7) | | 92 (14.5) | |  |
| 2 to 4 | 5717 (48.1) | | | | | 1148 (55.8) | 5859 (49.1) | | | 1006 (50.0) | | 5944 (49.0) | | | 921 (50.8) | | | 5760 (48.9) | | 1105 (51.3) | | | 5798 (48.8) | | 1067 (51.9) | | | 6557 (49.3) | | 308 (48.7) | |  |
| Missing | 43 (0.4) | | | | | 4 (0.2) | 44 (0.4) | | | 3 (0.1) | | 47 (0.4) | | | 0 (0.0) | | | 39 (0.3) | | 8 (0.4) | | | 44 (0.4) | | 3 (0.1) | | | 47 (0.4) | | 0 (0.0) | |  |
| Chi square | | | | ꭓ ^2^ (2)= 50.48; *p*<.001 | | | | | ꭓ ^2^ (2)= 2.79;  *p>.99* | | | | ꭓ ^2^ (2)= 1.70; *p>.99* | | | | ꭓ ^2^ (2)= 18.26; *p*<.001 | | | | | ꭓ ^2^ (2)= 11.52 ;  *p*=.017 | | | | | ꭓ ^2^ (2)= 1.33;  *p>.99* | | | | | |
| Depressed mood |  | | | |  | | |  | | |  | | |  | |  | | |  | |  | | |  | |  | | |  | |  |  |
| 0 | 4794 (40.4) | | | | | 852 (41.4) | 4,873 (40.9) | | | 773 (38.4) | | 4946 (40.8) | | | 700 (38.6) | | | 4759 (40.4) | | 887 (41.2) | | | 4884 (41.1) | | 762 (37.0) | | | 5378 (40.4) | | 268 (42.3) | |  |
| 1 | 3376 (28.4) | | | | | 625 (30.4) | 3401 (28.5) | | | 600 (29.8) | | 3456 (28.5) | | | 545 (30.1) | | | 3359 (28.5) | | 642 (29.8) | | | 3363 (28.3) | | 638 (31.0) | | | 3828 (28.8) | | 173 (27.3) | |  |
| 2 to 4 | 3690 (31.1) | | | | | 576 (28.0) | 3628 (30.4) | | | 638 (31.7) | | 3699 (30.5) | | | 567 (31.3) | | | 3647 (31.0) | | 619 (28.8) | | | 3612 (30.4) | | 654 (31.8) | | | 4074 (30.6) | | 192 (30.3) | |  |
| Missing | 14 (0.1) | | | | | 6 (0.3) | 19 (0.2) | | | 1 (0.0) | | 20 (0.2) | | | 0 (0.0) | | | 16 (0.1) | | 4 (0.2) | | | 17 (0.1) | | 3 (0.1) | | | 20 (0.2) | | 0 (0.0) | |  |
| Chi square | | | | ꭓ ^2^ (2)= 8.21;  *p* =.088 | | | | | ꭓ ^2^ (2)= 4.48;  *p* =.589 | | | | ꭓ ^2^ (2)= 3.48;  *p* =.963 | | | | ꭓ ^2^ (2)= 4.27;  *p* =.649 | | | | | ꭓ ^2^ (2)= 12.74;  *p*=.011 | | | | | ꭓ ^2^ (2)= 1.00;  *p>.99* | | | | | |
| Other mental problems |  | | | |  | | |  | | |  | | |  | |  | | |  | |  | | |  | |  | | |  | |  |  |
| 0 | 3195 (26.9) | | | | | 571 (27.7) | 3229 (27.1) | | | 537 (26.7) | | 3272 (27.0) | | | 494 (27.3) | | | 3179 (27.0) | | 587 (27.3) | | | 3237 (27.3) | | 529 (25.7) | | | 3591 (27.0) | | 175 (27.6) | |  |
| 1 | 2,401 (20.2) | | | | | 418 (20.3) | 2442 (20.5) | | | 377 (18.7) | | 2,476 (20.4) | | | 343 (18.9) | | | 2383 (20.2) | | 436 (20.3) | | | 2,443 (20.6) | | 376 (18.3) | | | 2701 (20.3) | | 118 (18.6) | |  |
| 2 to 4 | 6218 (52.4) | | | | | 1,063 (51.6) | 6,188 (51.9) | | | 1,093 (54.3) | | 6314 (52.1) | | | 967 (53.4) | | | 6157 (52.3) | | 1124 (52.2) | | | 6135 (51.7) | | 1146 (55.7) | | | 6943 (52.2) | | 338 (53.4) | |  |
| Missing | 60 (0.5) | | | | | 7 (0.3) | 62 (0.5) | | | 5 (0.2) | | 59 (0.5) | | | 8 (0.4) | | | 62 (0.5) | | 5 (0.2) | | | 61 (0.5) | | 6 (0.3) | | | 65 (0.5) | | 2 (0.3) | |  |
| Chi square | | | | ꭓ ^2^ (2)=0.63;  *p>.99* | | | | | ꭓ ^2^ (2)= 4.60;  *p* =.550 | | | | ꭓ ^2^ (2)=2.28;  *p>.99* | | | | ꭓ ^2^ (2)= 0.04;  *p>.99* | | | | | ꭓ ^2^ (2)= 11.58;  *p* =.017 | | | | | ꭓ ^2^ (2)= 1.08;  *p>.99* | | | | | |
| Relationship Problems | | | |  | | | | |  | | | |  | | | |  | | | | |  | | | | |  | | | | | |
| 0 | 4591 (38.7) | | | | | 748 (36.3) | 4619 (38.7) | | | 720 (35.8) | | 4665 (38.5) | | | 674 (37.2) | | | 4579 (38.9) | | 760 (35.3) | | | 4610 (38.8) | | 729 (35.4) | | | 5097 (38.3) | | 242 (38.2) | |  |
| 1 | 3082 (26.0) | | | | | 549 (26.7) | 3098 (26.0) | | | 533 (26.5) | | 3167 (26.1) | | | 464 (25.6) | | | 3043 (25.8) | | 588 (27.3) | | | 3076 (25.9) | | 555 (27.0) | | | 3467 (26.1) | | 164 (25.9) | |  |
| 2 to 4 | 4126 (34.7) | | | | | 749 (36.4) | 4136 (34.7) | | | 739 (36.7) | | 4213 (34.8) | | | 662 (36.5) | | | 4088 (34.7) | | 787 (36.6) | | | 4117 (34.7) | | 758 (36.8) | | | 4652 (35.0) | | 223 (35.2) | |  |
| Missing | 75 (0.6) | | | | | 13 (0.6) | 68 (0.6) | | | 20 (1.0) | | 76 (0.6) | | | 12 (0.7) | | | 71 (0.6) | | 17 (0.8) | | | 73 (0.6) | | 15 (0.7) | | | 84 (0.6) | | 4 (0.6) | |  |
| Chi square | | | | ꭓ ^2^ (2)= 4.17;  *p* =.688 | | | | | ꭓ ^2^ (2)= 6.13;  *p* =.259 | | | | ꭓ ^2^ (2)= 2.27;  *p>.99* | | | | ꭓ ^2^ (2)= 9.37;  *p* =.050 | | | | | ꭓ ^2^ (2)= 8.40;  *p*=.083 | | | | | ꭓ ^2^ (2)= 0.02;  *p>.99* | | | | | |
| Daily living problems |  | | | |  | | |  | | |  | | |  | |  | | |  | |  | | |  | |  | | |  | |  |  |
| 0 | 6281 (52.9) | | | | | 910 (44.2) | 6249 (52.4) | | | 942 (46.8) | | 6,375 (52.6) | | | 816 (45.0) | | | 6216 (52.8) | | 975 (45.3) | | | 6258 (52.7) | | 933 (45.4) | | | 6895 (51.8) | | 296 (46.8) | |  |
| 1 | 2540 (21.4) | | | | | 500 (24.3) | 2544 (21.3) | | | 496 (24.7) | | 2605 (21.5) | | | 435 (24.0) | | | 2538 (21.5) | | 502 (23.3) | | | 2529 (21.3) | | 511 (24.8) | | | 2893 (21.8) | | 147 (23.2) | |  |
| 2 to 4 | 2978 (25.1) | | | | | 642 (31.2) | 3057 (25.6) | | | 563 (28.0) | | 3064 (25.3) | | | 556 (30.7) | | | 2956 (25.1) | | 664 (30.9) | | | 3023 (25.5) | | 597 (29.0) | | | 3433 (25.8) | | 187 (29.5) | |  |
| Missing | 75 (0.6) | | | | | 7 (0.3) | 71 (0.6) | | | 11 (0.5) | | 77 (0.6) | | | 5 (0.3) | | | 71 (0.6) | | 11 (0.5) | | | 66 (0.6) | | 16 (0.8) | | | 79 (0.6) | | 3 (0.5) | |  |
| Chi square | | | | ꭓ ^2^ (2)= 57.47 (2) ;*p*<.001 | | | | | ꭓ ^2^ (2)= 22.73 (2) ;*p*<.001) | | | | ꭓ ^2^ (2)= 39.64 *p*<.001 | | | | ꭓ ^2^ (2)= 45.49; *p*<.001 | | | | | ꭓ ^2^ (2)= 37.04;  *p*<.001 | | | | | ꭓ ^2^ (2)= 6.85; *p* =.182 | | | | | |
| Living condition problems |  | | | |  | | |  | | |  | | |  | |  | | |  | |  | | |  | |  | | |  | |  |  |
| 0 | 7240 (61.0) | | | | | 1,228 (59.6) | 7249 (60.8) | | | 1219 (60.6) | | 7373 (60.8) | | | 1095 (60.4) | | | 7218 (61.3) | | 1250 (58.1) | | | 7272 (61.2) | | 1196 (58.1) | | | 8086 (60.8) | | 382 (60.3) | |  |
| 1 | 1916 (16.1) | | | | | 361 (17.5) | 1945 (16.3) | | | 332 (16.5) | | 1978 (16.3) | | | 299 (16.5) | | | 1908 (16.2) | | 369 (17.1) | | | 1928 (16.2) | | 349 (17.0) | | | 2168 (16.3) | | 109 (17.2) | |  |
| 2 to 4 | 2386 (20.1) | | | | | 402 (19.5) | 2386 (20.0) | | | 402 (20.0) | | 2432 (20.1) | | | 356 (19.6) | | | 2326 (19.7) | | 462 (21.5) | | | 2348 (19.8) | | 440 (21.4) | | | 2,671 (20.1) | | 117 (18.5) | |  |
| Missing | 332 (2.8) | | | | | 68 (3.3) | 341 (2.9) | | | 59 (2.9) | | 338 (2.8) | | | 62 (3.4) | | | 329 (2.8) | | 71 (3.3) | | | 328 (2.8) | | 72 (3.5) | | | 375 (2.8) | | 25 (3.9) | |  |
| Chi square | | | | ꭓ ^2^ (2)= 2.86;  *p>.99* | | | | | ꭓ ^2^ (2)= 0.05;  *p>.99* | | | | ꭓ ^2^ (2)= 0.15;  *p>.99* | | | | ꭓ ^2^ (2)= 6.72;  *p* = .193 | | | | | ꭓ ^2^ (2)= 5.56;  *p* = .341 | | | | | ꭓ ^2^ (2)= 1.04;  *p>.99* | | | | | |
| Occupational problems | |  | | |  | | |  | | |  | | |  | |  | | |  | |  | | |  | |  | | |  | |  |  |
| 0 | 5469 (46.1) | | | | | 874 (42.4) | 5491 (46.1) | | | 852 (42.3) | | 5555 (45.8) | | | 788 (43.5) | | | 5434 (46.1) | | 909 (42.2) | | | 5443 (45.8) | | 900 (43.8) | | | 6,061 (45.6) | | 282 (44.5) | |  |
| 1 | 2640 (22.2) | | | | | 483 (23.5) | 2633 (22.1) | | | 490 (24.4) | | 2689 (22.2) | | | 434 (24.0) | | | 2616 (22.2) | | 507 (23.6) | | | 2655 (22.4) | | 468 (22.8) | | | 2959 (22.2) | | 164 (25.9) | |  |
| 2 to 4 | 3493 (29.4) | | | | | 641 (31.1) | 3518 (29.5) | | | 616 (30.6) | | 3587 (29.6) | | | 547 (30.2) | | | 3460 (29.4) | | 674 (31.3) | | | 3,503 (29.5) | | 631 (30.7) | | | 3963 (29.8) | | 171 (27.0) | |  |
| Missing | 272 (2.3) | | | | | 61 (3.0) | 279 (2.3) | | | 54 (2.7) | | 290 (2.4) | | | 43 (2.4) | | | 271 (2.3) | | 62 (2.9) | | | 275 (2.3) | | 58 (2.8) | | | 317 (2.4) | | 16 (2.5) | |  |
| Chi-square | | | | ꭓ ^2^ (2)= 7.90;  *p* = .105 | | | | | ꭓ ^2^ (2)= 9.89; *p* =.039 | | | | ꭓ ^2^ (2)= 4.28;  *p* =.649 | | | | ꭓ ^2^ (2)= 9.83;  *p* =.039 | | | | | ꭓ ^2^ (2)= 2.57; *p>.99* | | | | | ꭓ ^2^ (2)= 5.33;  *p*=.380 | | | | | |

Note. T-tests were conducted between the HoNOS scores of individuals with and without each ICD-10 disorder. Chi-square tests were conducted between each HoNOS subscale and individuals with and without each ICD-10 disorder. *Percentages are shown by column.

**missing values not included in statistical analyses

Supplementary Table 4A to 4K. Multinomial regressions demonstrating associations between physical comorbidities and HoNOS subscales, unadjusted and adjusted for age and gender.

| **Table 4A. Chapter II Neoplastic disorders** | | | | | | |
| --- | --- | --- | --- | --- | --- | --- |
|  | Unadjusted model | | | Sex and age adjusted model | | |
|  | B (SE) | 95% CI | p-value | B (SE) | 95% CI | p-value |
| **Problem drinking** |  |  |  |  |  |  |
| 0 (reference category) |  |  |  |  |  |  |
| 1 | -0.400 (0.149) | -0.692 – -0.108 | *p*<.001 | -0.121 (0.152) | -0.418 – 0.177 | *p*= .427 |
| 2 – 4 (most severe) | -0.292 (0.116) | -0.520 - -0.065 | *p*= .012 | 0.024 (0.120) | -0.211 – 0.259 | *p*= .843 |
| **Physical Illness** |  |  |  |  |  |  |
| 0 (reference category) |  |  |  |  |  |  |
| 1 | 0.349 (0.108) | 0.137 – 0.560 | *p*<.001 | 0.103 (0.112) | -0.116 – 0.322 | *p*= .356 |
| 2 – 4 (most severe) | 0.706 (0.087) | 0.535 – 0.877 | *p*<.001 | 0.350 (0.097) | 0.160 – 0.540 | *p*<.001 |
| **Depressed mood** |  |  |  |  |  |  |
| 0 (reference category) |  |  |  |  |  |  |
| 1 | 0.261 (0.092) | 0.081 – 0.442 | *p*<.001 | 0.303 (0.093) | 0.121 – 0.484 | *p*<.001 |
| 2 – 4 (most severe) | 0.086 (0.094) | -0.098 – 0.271 | *p*= .360 | 0.162 (0.096) | -0.025 – 0.350 | *p*= 0.090 |
| Table 4B. Chapter III Haematological Disorders | | | | | | |
|  | Unadjusted model | | | Sex and age adjusted model | | |
|  | B (SE) | 95% CI | p-value | B (SE) | 95% CI | p-value |
| **Agitated behaviours** |  |  |  |  |  |  |
| 0 (reference category) |  |  |  |  |  |  |
| 1 | 0.0069 (0.102) | -0.194 – 0.208 | *p*= .946 | 0.130 (0.103) | -0.188 – 0.214 | *p*= .896 |
| 2 – 4 (most severe) | 0.208 (0.094) | 0.023 – 0.393 | *p*= .027 | 2.27 (0.094) | 0.030 – 0.400 | *p*= .023 |
| **Physical Illness** |  |  |  |  |  |  |
| 0 (reference category) |  |  |  |  |  |  |
| 1 | -0.131 (0.122) | -0.370 – 0.107 | *p*= .280 | 0.021 (0.125) | -0.224 – 0.265 | *p*<.001 |
| 2 – 4 (most severe) | 0.206 (0.096) | 0.017 – 0.394 | *p*= .032 | 0.468 (0.106) | 0.261 – 0.676 | *p*<.001 |
| **Hallucinations** |  |  |  |  |  |  |
| 0 (reference category) |  |  |  |  |  |  |
| 1 | 0.462 (0.120) | 0.226 – 0.699 | *p*<.001 | 0.494 (0.121) | 0.257 – 0.731 | *p*<.001 |
| 2 – 4 (most severe) | 0.369 (0.094) | 0.184 – 0.553 | *p*<.001 | 0.420 (0.095) | 0.235 – 0.605 | *p*<.001 |
| **Depressed mood** |  |  |  |  |  |  |
| 0 (reference category) |  |  |  |  |  |  |
| 1 | 0.172 (0.093) | -0.011 – 0.355 | *p*= .066 | 0.146 (0.094) | -0.038 – 0.329 | *p*= .120 |
| 2 – 4 (most severe) | -0.188 (0.100) | -0.385 – 0.097 | *p*= .062 | -0.273 (0.102) | -0.472 – -0.074 | *p*<.001 |
| **Living condition problems** |  |  |  |  |  |  |
| 0 (reference category) |  |  |  |  |  |  |
| 1 | 0.051 (0.114) | -0.172 – 0.273 | *p*= .657 | 0.111 (0.114) | -0.112 – 0.335 | *p*= .329 |
| 2 – 4 (most severe) | 0.285 (0.098) | 0.094 - 0.476 | *p*<.001 | 0.328 (0.098) | 0.136 – 0.520 | *p*<.001 |
| **Occupational problems** |  |  |  |  |  |  |
| 0 (reference category) |  |  |  |  |  |  |
| 1 | 0.267 (0.103) | 0.066 - 0.469 | *p*<.001 | 0.288 (0.103) | 0.085 – 0.490 | *p*<.001 |
| 2 – 4 (most severe) | 0.279 (0.095) | 0.094 – 0.465 | *p*<.001 | 0.320 (0.095) | 0.134 – 0.506 | *p*<.001 |

| **Table 4C. Chapter IV Endocrine Disorders** | | | | | | |
| --- | --- | --- | --- | --- | --- | --- |
|  | Unadjusted model | | | Sex and age adjusted model | | |
|  | B (SE) | 95% CI | p-value | B (SE) | 95% CI | p-value |
| **Agitated behaviours** |  |  |  |  |  |  |
| 0 (reference category) |  |  |  |  |  |  |
| 1 | 0.130 (0.061) | 0.0096 – 0.250 | *p*<.001 | 0.132 (0.061) | 0.012 – 0.252 | *p*<.001 |
| 2 – 4 (most severe) | 0.116 (0.060) | -0.0020 – 0.234 | *p*<.001 | 0.117 (0.060) | -0.00093 – 0.235 | *p*<.001 |
| **Cognitive Disorders** |  |  |  |  |  |  |
| 0 (reference category) |  |  |  |  |  |  |
| 1 | 0.127 (0.063) | 0.0038 – 0.249 | *p*<.001 | 0.153 (0.063) | 0.029 – 0.276 | *p*<.001 |
| 2 – 4 (most severe) | 0.359 (0.064) | 0.234 – 0.483 | *p*<.001 | 0.389 (0.064) | 0.263 – 0.515 | *p*<.001 |
| **Physical illness** |  |  |  |  |  |  |
| 0 (reference category) |  |  |  |  |  |  |
| 1 | 0.303 (0.068) | 0.170 – 0.435 | *p*<.001 | 0.377 (0.070) | 0.241 – 0.514 | *p*<.001 |
| 2 – 4 (most severe) | 0.259 (0.061) | 0.139 – 0.378 | *p*<.001 | 0.410 (0.067) | 0.279 – 0.540 | *p*<.001 |
| **Hallucinations** |  |  |  |  |  |  |
| 0 (reference category) |  |  |  |  |  |  |
| 1 | 0.153 (0.076) | 0.005 – 0.302 | *p*= .043 | 0.158 (0.076) | 0.009 – 0.307 | *p*= .038 |
| 2 – 4 (most severe) | 0.172 (0.056) | 0.062 – 0.281 | *p*<.001 | 0.185 (0.056) | 0.075 – 0.295 | *p*<.001 |
| **Relationship problems** |  |  |  |  |  |  |
| 0 (reference category) |  |  |  |  |  |  |
| 1 | 0.008 (0.064) | -0.118 – 0.135 | *p*= .896 | 0.010 (0.064) | -0.116 – 0.137 | *p*= .873 |
| 2 – 4 (most severe) | 0.166 (0.058) | 0.053 – 0.279 | *p*<.001 | 0.170 (0.058) | 0.057 – 0.283 | *p*<.001 |
| **Daily living problems** |  |  |  |  |  |  |
| 0 (reference category) |  |  |  |  |  |  |
| 1 | 0.249 (0.063) | 0.126 – 0.373 | *p*<.001 | 0.265 (0.063) | 0.141 – 0.389 | *p*<.001 |
| 2 – 4 (most severe) | 0.359 (0.058) | 0.245– 0.474 | *p*<.001 | 0.401 (0.059) | 0.285 – 0.517 | *p*<.001 |
| **Occupational problems** |  |  |  |  |  |  |
| 0 (reference category) |  |  |  |  |  |  |
| 1 | 0.146 (0.064) | 0.021 - 0.272 | *p*= .022 | 0.288 (0.103) | 0.085 – 0.490 | *p*<.001 |
| 2 – 4 (most severe) | 0.162 (0.058) | 0.047 – 0.277 | *p*<.001 | 0.320 (0.095) | 0.134 – 0.506 | *p*<.001 |
| **Table 4D. Chapter VI Neurological Disorders** | | | | | | |
|  | Unadjusted model | | | Sex and age adjusted model | | |
|  | B (SE) | 95% CI | p-value | B (SE) | 95% CI | p-value |
| **Self Injury** |  |  |  |  |  |  |
| 0 (reference category) |  |  |  |  |  |  |
| 1 | -0.0095(0.065) | -0.137 – 0.118 | *p*= .884 | -0.0028(0.065) | -0.131 – 0.125 | *p*= .966 |
| 2 – 4 (most severe) | 0.157 (0.069) | 0.023 – 0.292 | *p*<.001 | 0.172 (0.069) | 0.037 – 0.307 | *p*<.001 |
| **Cognitive Disorders** |  |  |  |  |  |  |
| 0 (reference category) |  |  |  |  |  |  |
| 1 | 0.135 (0.045) | 0.047 – 0.222 | *p*<.001 | 0.131 (0.045) | 0.043 – 0.219 | *p*<.001 |
| 2 – 4 (most severe) | 0.174(0.048) | 0.080 – 0.269 | *p*<.001 | 0.169 (0.048) | 0.074 – 0.264 | *p*<.001 |
| **Physical illness** |  |  |  |  |  |  |
| 0 (reference category) |  |  |  |  |  |  |
| 1 | 0.260 (0.050) | 0.161 – 0.358 | *p*<.001 | 0.261 (0.052) | 0.160 – 0.362 | *p*<.001 |
| 2 – 4 (most severe) | 0.302 (0.044) | 0.214 – 0.389 | *p*<.001 | 0.312 (0.049) | 0.216 – 0.407 | *p*<.001 |
| **Hallucinations** |  |  |  |  |  |  |
| 0 (reference category) |  |  |  |  |  |  |
| 1 | 0.121 (0.054) | 0.015 – 0.227 | *p*= .025 | 0.136 (0.054) | 0.009 – 0.307 | *p*= .013 |
| 2 – 4 (most severe) | 0.127 (0.040) | 0.050 – 0.205 | *p*<.001 | 0.132 (0.040) | 0.075 – 0.295 | *p*<.001 |
| **Depressed mood** |  |  |  |  |  |  |
| 0 (reference category) |  |  |  |  |  |  |
| 1 | 0.110 (0.043) | 0.026 – 0.195 | *p*= .011 | 0.113 (0.043) | 0.028 – 0.198 | *p*<.001 |
| 2 – 4 (most severe) | 9.71 x 10^-3^ (0.043) | -0.074 – 0.094 | *p*= .820 | 9.73 x 10^-3^  (0.043) | -0.075 – 0.094 | *p*= .822 |
| **Daily living problems** |  |  |  |  |  |  |
| 0 (reference category) |  |  |  |  |  |  |
| 1 | 0.210 (0.045) | 0.122- 0.299 | *p*<.001 | 0.209 (0.045) | 0.121 – 0.298 | *p*<.001 |
| 2 – 4 (most severe) | 0.188 (0.043) | 0.105 – 0.272 | *p*<.001 | 0.182 (0.043) | 0.097 – 0.267 | *p*<.001 |

| **Table 4E. Chapter VII Eye/ Adnexal disorders** | | | | | | |
| --- | --- | --- | --- | --- | --- | --- |
|  | Unadjusted model | | | Sex and age adjusted model | | |
|  | B (SE) | 95% CI | p-value | B (SE) | 95% CI | p-value |
| **Cognitive Disorders** |  |  |  |  |  |  |
| 0 (reference category) |  |  |  |  |  |  |
| 1 | 0.259 (0.070) | 0.123 – 0.395 | *p*<.001 | 0.263 (0.070) | 0.126 – 0.401 | *p*<.001 |
| 2 – 4 (most severe) | 0.287 (0.074) | 0.141 – 0.432 | *p*<.001 | 0.291 (0.075) | 0.144 – 0.438 | *p*<.001 |
| **Physical illness** |  |  |  |  |  |  |
| 0 (reference category) |  |  |  |  |  |  |
| 1 | 0.169 (0.081) | 0.011 – 0.328 | *p*<.001 | 0.228 (0.083) | 0.065 – 0.391 | *p*<.001 |
| 2 – 4 (most severe) | 0.413 (0.067) | 0.281 – 0.545 | *p*<.001 | 0.491 (0.075) | 0.344 – 0.638 | *p*<.001 |
| **Hallucinations** |  |  |  |  |  |  |
| 0 (reference category) |  |  |  |  |  |  |
| 1 | 0.354 (0.085) | 0.186 – 0.521 | *p*<.001 | 0.354 (0.085) | 0.187 – 0.522 | *p*<.001 |
| 2 – 4 (most severe) | 0.271 (0.065) | 0.144 – 0.399 | *p*<.001 | 0.272 (0.065) | 0.144 – 0.401 | *p*<.001 |
| **Daily living problems** |  |  |  |  |  |  |
| 0 (reference category) |  |  |  |  |  |  |
| 1 | 0.350 (0.070) | 0.212 – 0.487 | *p*<.001 | 0.353 (0.070) | 0.215 – 0.491 | *p*<.001 |
| 2 – 4 (most severe) | 0.262 (0.068) | 0.129 – 0.395 | *p*<.001 | 0.268 (0.069) | 0.133 – 0.403 | *p*<.001 |

| **Table 4F. Chapter IX Circulatory Disorders** | | | | | | |
| --- | --- | --- | --- | --- | --- | --- |
|  | Unadjusted model | | | Sex and age adjusted model | | |
|  | B (SE) | 95% CI | p-value | B (SE) | 95% CI | p-value |
| **Agitated behaviour** |  |  |  |  |  |  |
| 0 (reference category) |  |  |  |  |  |  |
| 1 | 0.097 (0.059) | -0.020 – 0.213 | *p*= .103 | 0.089 (0.059) | -0.028 – 0.206 | *p*= 0.135 |
| 2 – 4 (most severe) | 0.151 (0.057) | 0.038 – 0.263 | *p*<.001 | 0.152 (0.058) | 0.039 – 0.265 | *p*<.001 |
| **Cognitive disorders** |  |  |  |  |  |  |
| 0 (reference category) |  |  |  |  |  |  |
| 1 | 0.356 (0.058) | 0.242 – 0.471 | *p*<.001 | 0.305 (0.059) | 0.190 – 0.421 | *p*<.001 |
| 2 – 4 (most severe) | 0.403 (0.062) | 0.282 – 0.525 | *p*<.001 | 0.344 (0.063) | 0.221 – 0.467 | *p*<.001 |
| **Physical illness** |  |  |  |  |  |  |
| 0 (reference category) |  |  |  |  |  |  |
| 1 | 0.383 (0.066) | 0.253 – 0.513 | *p*<.001 | 0.292 (0.069) | 0.157 – 0.426 | *p*<.001 |
| 2 – 4 (most severe) | 0.646 (0.056) | 0.536 – 0.756 | *p*<.001 | 0.486 (0.063) | 0.364 – 0.609 | *p*<.001 |
| **Hallucinations** |  |  |  |  |  |  |
| 0 (reference category) |  |  |  |  |  |  |
| 1 | 0.260 (0.075) | 0.114 – 0.406 | *p*<.001 | 0.276 (0.075) | 0.129 – 0.423 | *p*<.001 |
| 2 – 4 (most severe) | 0.388 (0.055) | 0.281 – 0.495 | *p*<.001 | 0.368 (0.055) | 0.260 – 0.476 | *p*<.001 |
| **Daily living condition problems** |  |  |  |  |  |  |
| 0 (reference category) |  |  |  |  |  |  |
| 1 | 0.303 (0.060) | 0.185 – 0.421 | *p*<.001 | 0.275 (0.061) | 0.156 – 0.394 | *p*<.001 |
| 2 – 4 (most severe) | 0.396 (0.056) | 0.286 – 0.506 | *p*<.001 | 0.321 (0.057) | 0.209 – 0.433 | *p*<.001 |
| **Occupational problems** |  |  |  |  |  |  |
| 0 (reference category) |  |  |  |  |  |  |
| 1 | 0.135 (0.061) | 0.015 – 0.256 | *p*<.001 | 0.146 (0.062) | 0.025 – 0.267 | *p*<.001 |
| 2 – 4 (most severe) | 0.138 (0.056) | 0.027 – 0.248 | *p*<.001 | 0.142 (0.057) | 0.031 – 0.253 | *p*<.001 |

| **Table 4G. Chapter X Respiratory disorders** | | | | | | |
| --- | --- | --- | --- | --- | --- | --- |
|  | Unadjusted model | | | Sex and age adjusted model | | |
|  | B (SE) | 95% CI | p-value | B (SE) | 95% CI | p-value |
| **Agitated behaviour** |  |  |  |  |  |  |
| 0 (reference category) |  |  |  |  |  |  |
| 1 | 0.075 (0.061) | -0.044 – 0.194 | *p*=.214 | 0.081 (0.060) | -0.038 – 0.200 | *p*<.001 |
| 2 – 4 (most severe) | 0.227 (0.057) | 0.115 – 0.340 | *p*<.001 | 0.231 (0.055) | 0.118 – 0.344 | *p*<.001 |
| **Self injury** |  |  |  |  |  |  |
| 0 (reference category) |  |  |  |  |  |  |
| 1 | 0.233 (0.083) | 0.071 – 0.395 | *p*<.001 | 0.196 (0.083) | 0.034 – 0.359 | *p*= .018 |
| 2 – 4 (most severe) | 0.216 (0.090) | 0.040 – 0.391 | *p*= .016 | 0.177 (0.090) | 0.0001 – 0.353 | *p*=.050 |
| **Problem drinking** |  |  |  |  |  |  |
| 0 (reference category) |  |  |  |  |  |  |
| 1 | 0.165 (0.080) | 0.00835 – 0.321 | *p*<.001 | 0.144 (0.081) | -0.015 – 0.303 | *p*= .076 |
| 2 – 4 (most severe) | 0.229 (0.064) | 0.103 – 0.355 | *p*<.001 | 0.207 (0.067) | 0.076 – 0.338 | *p*<.001 |
| **Cognitive problems** |  |  |  |  |  |  |
| 0 (reference category) |  |  |  |  |  |  |
| 1 | 0.156 (0.060) | 0.039 – 0.274 | *p*<.001 | 0.201 (0.061) | 0.082 – 0.320 | *p*<.001 |
| 2 – 4 (most severe) | 0.263 (0.063) | 0.139 – 0.386 | *p*<.001 | 0.313 (0.064) | 0.188 – 0.438 | *p*<.001 |
| **Physical illness** |  |  |  |  |  |  |
| 0 (reference category) |  |  |  |  |  |  |
| 1 | 0.162 (0.067) | 0.030 – 0.295 | *p*<.001 | 0.281 (0.070) | 0.145 – 0.418 | *p*<.001 |
| 2 – 4 (most severe) | 0.215 (0.059) | 0.098 – 0.331 | *p*<.001 | 0.414 (0.065) | 0.287 – 0.542 | *p*<.001 |
| **Relationship problems** |  |  |  |  |  |  |
| 0 (reference category) |  |  |  |  |  |  |
| 1 | 0.097 (0.062) | -0.024 – 0.218 | *p*= .117 | 0.101 (0.062) | -0.020 – 0.222 | *p*= .101 |
| 2 – 4 (most severe) | 0.134 (0.057) | 0.023 – 0.245 | *p*<.001 | 0.143 (0.057) | 0.032 – 0.254 | *p*<.001 |
| **Daily living condition problems** |  |  |  |  |  |  |
| 0 (reference category) |  |  |  |  |  |  |
| 1 | 0.260 (0.060) | 0.142 - 0.378 | *p*<.001 | 0.288 (0.060) | 0.169 – 0.406 | *p*<.001 |
| 2 – 4 (most severe) | 0.198 (0.058) | 0.085 – 0.311 | *p*<.001 | 0.259 (0.059) | 0.144 – 0.374 | *p*<.001 |
| **Occupational problems** |  |  |  |  |  |  |
| 0 (reference category) |  |  |  |  |  |  |
| 1 | 0.182 (0.061) | 0.062 - 0.302 | *p*<.001 | 0.186 (0.062) | 0.065 – 0.307 | *p*<.001 |
| 2 – 4 (most severe) | 0.122 (0.057) | 0.010 – 0.234 | *p*<.001 | 0.133 (0.057) | 0.021 – 0.246 | *p*<.001 |

| **Table 4H. Chapter XI Digestive Disorders** | | | | | | |
| --- | --- | --- | --- | --- | --- | --- |
|  | Unadjusted model | | | Sex and age adjusted model | | |
|  | B (SE) | 95% CI | p-value | B (SE) | 95% CI | p-value |
| **Agitated behaviour** |  |  |  |  |  |  |
| 0 (reference category) |  |  |  |  |  |  |
| 1 | 0.113 (0.062) | -0.010-0.235 | *p*<.001 | 0.108 (0.063) | -0.014 – 0.231 | *p=*0.083 |
| 2 – 4 (most severe) | 0.129 (0.061) | 0.010 – 0.248 | *p*<.001 | 0.130 (0.061) | 0.010 – 0.249 | *p=*0.033 |
| **Self injury** |  |  |  |  |  |  |
| 0 (reference category) |  |  |  |  |  |  |
| 1 | 0.092 (0.090) | -0.083 – 0.268 | *p=*.303 | 0.124 (0.090) | -0.053 – 0.300 | *p=*.171 |
| 2 – 4 (most severe) | 0.200 (0.093) | 0.017 – 0.383 | *p=.*032 | 0.241 (0.094) | 0.057 – 0.425 | *p=.*010 |
| **Cognitive problems** |  |  |  |  |  |  |
| 0 (reference category) |  |  |  |  |  |  |
| 1 | 0.130 (0.063) | 0.0068 – 0.254 | *p*= .039 | 0.104 (0.063) | -0.020 – 0.228 | *p*= .101 |
| 2 – 4 (most severe) | 0.229 (0.066) | 0.0995 – 0.359 | *p*<.001 | 0.199 (0.067) | 0.068 – 0.330 | *p*<.001 |
| **Physical illness** |  |  |  |  |  |  |
| 0 (reference category) |  |  |  |  |  |  |
| 1 | 0.295 (0.070) | 0.157 – 0.433 | *p*<.001 | 0.269 (0.072) | 0.128 – 0.411 | *p*<.001 |
| 2 – 4 (most severe) | 0.506 (0.060) | 0.389 – 0.623 | *p*<.001 | 0.471 (0.066) | 0.341 – 0.600 | *p*<.001 |
| **Daily living condition problems** |  |  |  |  |  |  |
| 0 (reference category) |  |  |  |  |  |  |
| 1 | 0.262 (0.064) | 0.137 - 0.387 | *p*<.001 | 0.249 (0.064) | 0.124 – 0.375 | *p*<.001 |
| 2 – 4 (most severe) | 0.345 (0.059) | 0.229 – 0.461 | *p*<.001 | 0.313 (0.060) | 0.196 – 0.431 | *p*<.001 |

| **Table 4I. Chapter XII Dermatological disorders** | | | | | | |
| --- | --- | --- | --- | --- | --- | --- |
|  | Unadjusted model | | | Sex and age adjusted model | | |
|  | B (SE) | 95% CI | p-value | B (SE) | 95% CI | p-value |
| **Agitated behaviour** |  |  |  |  |  |  |
| 0 (reference category) |  |  |  |  |  |  |
| 1 | 0.122 (0.058) | 0.0088 – 0.236 | *p*= .035 | 0.121 (0.058) | 0.0074 – 0.235 | *p*= .037 |
| 2 – 4 (most severe) | 0.111 (0.057) | -0.000106 – 0.223 | *p*= .050 | 0.114 (0.057) | 0.0028 – 0.226 | *p*= .045 |
| **Cognitive problems** |  |  |  |  |  |  |
| 0 (reference category) |  |  |  |  |  |  |
| 1 | 0.215 (0.058) | 0.101 – 0.328 | *p*<.001 | 0.218 (0.058) | 0.103 – 0.332 | *p*<.001 |
| 2 – 4 (most severe) | 0.263 (0.061) | 0.142 – 0.384 | *p*<.001 | 0.265 (0.062) | 0.143 – 0.387 | *p*<.001 |
| **Physical illness** |  |  |  |  |  |  |
| 0 (reference category) |  |  |  |  |  |  |
| 1 | 0.211 (0.066) | 0.083 – 0.340 | *p*<.001 | 0.224 (0.068) | 0.092 – 0.358 | *p*<.001 |
| 2 – 4 (most severe) | 0.372 (0.056) | 0.262 – 0.483 | *p*<.001 | 0.395 (0.062) | 0.273 – 0.518 | *p*<.001 |
| **Hallucinations** |  |  |  |  |  |  |
| 0 (reference category) |  |  |  |  |  |  |
| 1 | 0.270 (0.070) | 0.132 – 0.408 | *p*<.001 | 0.291 (0.071) | 0.152 – 0.429 | *p*<.001 |
| 2 – 4 (most severe) | 0.189 (0.053) | 0.085 – 0.293 | *p*<.001 | 0.198 (0.053) | 0.094 – 0.303 | *p*<.001 |
| **Relationship problems** |  |  |  |  |  |  |
| 0 (reference category) |  |  |  |  |  |  |
| 1 | 0.154 (0.060) | 0.037 – 0.271 | *p*<.001 | 0.163 (0.060) | 0.046 – 0.280 | *p*<.001 |
| 2 – 4 (most severe) | 0.149 (0.055) | 0.040 – 0.257 | *p*<.001 | 0.157 (0.055) | 0.048 – 0.265 | *p*<.001 |
| **Daily living problems** |  |  |  |  |  |  |
| 0 (reference category) |  |  |  |  |  |  |
| 1 | 0.230 (0.060) | 0.112 - 0.347 | *p*<.001 | 0.235 (0.060) | 0.118 – 0.353 | *p*<.001 |
| 2 – 4 (most severe) | 0.358 (0.055) | 0.250 – 0.466 | *p*<.001 | 0.365 (0.056) | 0.256 – 0.475 | *p*<.001 |
| **Living condition problems** |  |  |  |  |  |  |
| 0 (reference category) |  |  |  |  |  |  |
| 1 | 0.111 (0.065) | -0.015 – 0.238 | *p*= .084 | 0.111 (0.065) | -0.016 – 0.238 | *p*= .086 |
| 2 – 4 (most severe) | 0.137 (0.059) | 0.020 – 0.253 | *p*= .021 | 0.150 (0.060) | 0.033 – 0.267 | *p*= .012 |
| **Occupational problems** |  |  |  |  |  |  |
| 0 (reference category) |  |  |  |  |  |  |
| 1 | 0.147 (0.060) | 0.029 - 0.266 | *p*= .015 | 0.160 (0.060) | 0.041 – 0.278 | *p*<.001 |
| 2 – 4 (most severe) | 0.152 (0.055) | 0.043 – 0.260 | *p*<.001 | 0.169 (0.055) | 0.060 – 0.278 | *p*<.001 |

| **Table 4J. Chapter XIII Musculoskeletal disorders** | | | | | | |
| --- | --- | --- | --- | --- | --- | --- |
|  | Unadjusted model | | | Sex and age adjusted model | | |
|  | B (SE) | 95% CI | p-value | B (SE) | 95% CI | p-value |
| **Agitated behaviour** |  |  |  |  |  |  |
| 0 (reference category) |  |  |  |  |  |  |
| 1 | 0.098 (0.060) | -0.019 – 0.215 | *p*= .101 | 0.096 (0.060) | -0.021 – 0.214 | *p*= .107 |
| 2 – 4 (most severe) | 0.205 (0.057) | 0.093 – 0.317 | *p*<.001 | 0.209 (0.057) | 0.097 – 0.321 | *p*<.001 |
| **Physical illness** |  |  |  |  |  |  |
| 0 (reference category) |  |  |  |  |  |  |
| 1 | 0.291 (0.067) | 0.160 – 0.422 | *p*<.001 | 0.306 (0.069) | 0.171 – 0.441 | *p*<.001 |
| 2 – 4 (most severe) | 0.554 (0.056) | 0.443 – 0.664 | *p*<.001 | 0.606 (0.062) | 0.484 – 0.728 | *p*<.001 |
| **Hallucinations** |  |  |  |  |  |  |
| 0 (reference category) |  |  |  |  |  |  |
| 1 | 0.172 (0.073) | 0.030 – 0.315 | *p*= .018 | 0.197 (0.073) | 0.054 – 0.340 | *p*<.001 |
| 2 – 4 (most severe) | 0.171 (0.054) | 0.066 – 0.276 | *p*<.001 | 0.180 (0.054) | 0.074 – 0.286 | *p*<.001 |
| **Depressed mood** |  |  |  |  |  |  |
| 0 (reference category) |  |  |  |  |  |  |
| 1 | 0.196 (0.058) | 0.082 – 0.310 | *p*<.001 | 0.199 (0.058) | 0.085 – 0.314 | *p*<.001 |
| 2 – 4 (most severe) | 0.148 (0.058) | 0.035 – 0.261 | *p*= .010 | 0.146 (0.058) | 0.032 – 0.260 | *p*= .012 |
| **Other mental disorders** |  |  |  |  |  |  |
| 0 (reference category) |  |  |  |  |  |  |
| 1 | -0.055 (0.073) | -0.198 – 0.087 | *p*= .444 | -0.045 (0.073) | -0.188 – 0.097 | *p*= .535 |
| 2 – 4 (most severe) | 0.134 (0.057) | 0.023 – 0.245 | *p*= .018 | 0.141 (0.057) | 0.029 – 0.253 | *p*= .0136 |
| **Relationship problems** |  |  |  |  |  |  |
| 0 (reference category) |  |  |  |  |  |  |
| 1 | 0.132 (0.061) | 0.012 – 0.251 | *p*= .031 | 0.143 (0.061) | 0.023 – 0.262 | *p*= .019 |
| 2 – 4 (most severe) | 0.151 (0.056) | 0.041 – 0.261 | *p*<.001 | 0.160 (0.056) | 0.050 – 0.271 | *p*<.001 |
| **Daily living disorders** |  |  |  |  |  |  |
| 0 (reference category) |  |  |  |  |  |  |
| 1 | 0.303 (0.060) | 0.186 - 0.420 | *p*<.001 | 0.305 (0.060) | 0.187 – 0.422 | *p*<.001 |
| 2 – 4 (most severe) | 0.278 (0.057) | 0.167 – 0.390 | *p*<.001 | 0.279 (0.058) | 0.167 – 0.392 | *p*<.001 |
| **Living condition problems** |  |  |  |  |  |  |
| 0 (reference category) |  |  |  |  |  |  |
| 1 | 0.094 (0.066) | -0.035 – 0.223 | *p*= .152 | 0.094 (0.066) | -0.036 -0.224 | *p*= .156 |
| 2 – 4 (most severe) | 0.129 (0.061) | 0.0099 – 0.247 | *p*= .034 | 0.144 (0.061) | 0.025 – 0.263 | *p*= .018 |

| **Table 4K. Chapter XIV Genitourinary Disorders** | | | | | | |
| --- | --- | --- | --- | --- | --- | --- |
|  | Unadjusted model | | | Sex and age adjusted model | | |
|  | B (SE) | 95% CI | p-value | B (SE) | 95% CI | p-value |
| **Physical illness** |  |  |  |  |  |  |
| 0 (reference category) |  |  |  |  |  |  |
| 1 | 0.436 (0.117) | 0.207 – 0.665 | *p*<.001 | 0.365 (0.121) | 0.128 – 0.602 | *p*<.001 |
| 2 – 4 (most severe) | 0.995 (0.090) | 0.819 – 1.172 | *p*<.001 | 0.889 (0.101) | 0.690 – 1.087 | *p*<.001 |

**Supplementary table 5.** Descriptive statistics for the sample of individuals with both SSD and BD diagnoses (N=749).

| **HoNOS Total Score (SD)** | **Total cohort with both diagnoses**  749  10.22 (6.39) | **No complex multimorbidity n(%)**  273 (36.4)  10.11 (6.25) | **Complex Multimorbidity n(%)**  476 (63.6)  10.28 (6.48) | **Statistical tests**  t= 40.97 (748); p <.001 |
| --- | --- | --- | --- | --- |
| **Age at diagnosis**  18 – 24  25 – 34  35 – 44  45 – 54  55 – 64  65 – 74  75 + | 220 (29.4)  224 (29.9)  152 (20.3)  85 (11.3)  38 (5.07)  24 (3.20)  6 (0.80) | 92 (33.7)  81 (29.7)  56 (20.5)  25 (9.16)  11 (4.03)  5 (1.83)  3 (1.10) | 128 (26.9)  143 (30.0)  96 (20.2)  60 (12.6)  27 (5.67)  19 (3.99)  3 (0.63) | ꭓ^2^(6) = 8.50; *p=.*204 |
| **Sex**  Female  Male | 401 (53.5)  348 (46.5) | 138 (50.5)  135 (49.5) | 263 (55.3)  213 (44.7) | ꭓ^2^(1)= 1.36 ;*p =.*244 |
| **Ethnicity**  British White  Black African  Black Caribbean  South Asian  Irish White  Chinese  Unknown | 213 (28.4)  134 (17.9)  52 (6.94)  24 (3.20)  9 (1.20)  5 (0.67)  312 (41.7) | 74 (27.1)  47 (17.2)  18 (6.59)  9 (3.30)  3 (1.10)  0 (0.00)  122 (44.7) | 139 (29.2)  87 (18.3)  34 (7.14)  15 (3.15)  6 (1.26)  5 (1.05)  190 (39.9) | ꭓ^2^(5)= 2.76 ;*p =.*737 |
| **IMD**  1  2  3  4  5  Unknown | 10 (1.34)  44 (5.87)  142 (19.0)  377 (50.3)  165 (22.0)  11 (1.47) | 5 (1.83)  16 (5.86)  45 (16.5)  147 (53.8)  56 (20.5)  4 (1.47) | 5 (1.05)  28 (5.88)  97 (20.4)  230 (48.3)  109 (22.9)  7 (1.47) | ꭓ^2^(4)= 3.68 ;*p =.*451 |
| **HONOS subscales** |  |  |  |  |
| **Agitated Behaviour**  0  1  2-4  Missing | 341 (45.5)  145 (19.4)  262 (35.0)  1 (0.13) | 123 (45.1)  53 (19.4)  97 (35.5)  0 (0.00) | 218 (86.1)  92 (19.3)  165 (34.7)  1 (0.21) | ꭓ ^2^ (2) =0.058; *p* =.971 |
| **Self-Injury**  0  1  2-4  Missing | 652 (87.0)  60 (8.01)  36 (4.81)  1 (0.134) | 242 (88.6)  18 (6.60)  12 (4.40)  1 (0.37) | 410 (86.1)  42 (8.82)  24 (5.04)  0 (0.00) | ꭓ ^2^ (2) =1.353; *p* =.509 |
| **Problem Drinking**  0  1  2-4  Missing | 551 (73.6)  69 (9.21)  119 (15.9)  10 (1.34) | 187 (68.5)  32 (11.7)  51 (18.7)  3 (1.10) | 364 (76.5)  37 (7.77)  68 (14.3)  7 (1.47) | ꭓ ^2^ (2) =6.536; *p* =.038 |
| **Cognitive Problems**  0  1  2-4  Missing | 493 (65.8)  133 (17.8)  122 (16.3)  1 (0.133) | 180 (65.9)  48 (17.6)  45 (16.5)  0 (0.00) | 313 (65.8)  85 (17.9)  77 (16.2)  1 (0.21) | ꭓ ^2^ (2) =0.017; *p* =.991 |
| **Physical Illness**  0  1  2-4  Missing | 567 (75.7)  86 (11.5)  92 (12.3)  4 (0.53) | 210 (76.9)  25 (9.16)  36 (13.2)  2 (0.73) | 357 (75.0)  61 (12.8)  56 (11.8)  2 (0.42) | ꭓ ^2^ (2) =2.392; *p* =.302 |
| **Hallucinations**  0  1  2-4  Missing | 250 (33.4)  133 (17.8)  361 (48.2)  4 (0.53) | 89 (32.6)  56 (20.5)  125 (45.8)  2 (0.73) | 161 (33.8)  77 (16.2)  236 (49.6)  2 (0.42) | ꭓ ^2^ (2) =2.429; *p* =.297 |
| **Depressed Mood**  0  1  2-4  Missing | 346 (46.2)  212 (28.3)  189 (25.3)  2 (0.27) | 132 (48.4)  76 (27.8)  65 (23.8)  0 (0.00) | 214 (45.0)  136 (28.6)  124 (25.1)  2 (0.42) | ꭓ ^2^ (2) =0.807; *p* =.668 |
| **Other Mental Problems**  0  1  2-4  Missing | 184 (24.6)  171 (22.8)  392 (52.3)  2 (0.27) | 61 (22.3)  75 (27.5)  136 (49.8)  1 (0.37) | 123 (25.8)  96 (20.2)  256 (53.8)  1 (0.21) | ꭓ ^2^ (2) =5.441; *p* =.066 |
| **Relationship Problems**  0  1  2-4  Missing | 309 (41.3)  194 (25.9)  243 (32.4)  3 (0.40) | 111 (40.7)  76 (27.8)  84 (30.8)  2 (0.73) | 199 (41.8)  118 (24.8)  159 (33.4)  1 (0.21) | ꭓ ^2^ (2) =1.027; *p=.*598 |
| **Daily Living Problems**  0  1  2-4  Missing | 426 (56.9)  167 (22.3)  150 (20.0)  6 (0.80) | 153 (56.0)  64 (23.4)  55 (20.1)  1 (0.37) | 273 (57.4)  103 (21.6)  95 (20.0)  5 (1.05) | ꭓ ^2^ (2) =0.300; *p* =.861 |
| **Living Conditions**  0  1  2-4  Missing | 462 (61.7)  119 (15.9)  129 (17.2)  39 (5.21) | 166 (60.8)  45 (16.5)  48 (17.6)  14 (5.13) | 296 (62.2)  74 (15.5)  81 (17.0)  25 (5.25) | ꭓ ^2^ (2) =0.181; *p* =.913 |
| **Occupational problems**  0  1  2-4  Missing | 363 (48.5)  187 (25.0)  181 (24.2)  18 (2.40) | 136 (49.8)  66 (24.2)  66 (24.2)  5 (1.83) | 227 (47.7)  121 (25.4)  115 (24.2)  13 (2.73) | ꭓ^2^(2)= 0.255 ;*p =.*880 |

Supplementary Table 6. Descriptive statistics for total HoNOS and subscales and comparisons between those with complex multimorbidity and without in the group of individuals with SSD diagnoses.

| **Patients with schizophrenia** | **Total** | **No Complex Multimorbidity** | **Complex Multimorbidity** | **Statistics** |
| --- | --- | --- | --- | --- |
| N (%) | 10554 | 3954 (37.5)* | 6600 (62.5)* |  |
| HONOS mean (SD) |  | 10.89 (6.17) | 10.99 (6.18) | t(10553) = 171.27 *p<.*001 |
| Agitated behaviour |  |  |  |  |
| 0 | 5705 (54.0) ** | 2136 (54.0)** | 3569 (54.1) |  |
| 1 | 2399 (22.7) ** | 900 (22.8)** | 1499 (22.7) |  |
| 2 to 4 | 2447 (23.2)** | 916 (23.2)** | 1531 (23.2) |  |
| Missing | 3 (0.028)** | 2 (0.051)** | 1 (0.015) | ꭓ^2^(2) = 0.00472; *p=.*998 |
| Self-injury |  |  |  |  |
| 0 | 9142 (86.6)** | 3453 (87.3)** | 5689 (86.2) |  |
| 1 | 755 (7.15)** | 275 (6.95)** | 480 (7.27) |  |
| 2 to 4 | 646 (6.12)** | 222 (5.61)** | 424 (6.43) |  |
| Missing | 11 (0.10)** | 4 (0.10)** | 7 (0.11) | ꭓ ^2^ (2) = 3.363; *p=*0.186 |
| Problem drinking |  |  | |  |
| 0 | 7893 (74.8)** | 2991 (75.6) | 4902 (74.3) |  |
| 1 | 953 (9.03)** | 342 (8.65) | 611 (9.26) |  |
| 2 to 4 | 1613 (15.28)** | 579 (14.6) | 1034 (15.7) |  |
| Missing | 95 (0.90)** | 42 (1.06) | 53 (0.80) | ꭓ ^2^ (2) =3.315; *p=* .191 |
| Cognitive problems |  |  | |  |
| 0 | 6226 (59.0)** | 2320 (58.7) | 3906 (59.2) |  |
| 1 | 2324 (22.0)** | 858 (21.7) | 1466 (22.2) |  |
| 2-4 | 1971 (18.7)** | 762 (19.3) | 1209 (18.3) |  |
| Missing | 33 (0.31)** | 14 (0.35) | 19 (0.29) | ꭓ ^2^ (2) =1.606; *p=*.448 |
| Physical illness |  |  | |  |
| 0 | 6684 (63.3)** | 2435 (61.6) | 4249 (64.9) |  |
| 1 | 1654 (15.7)** | 627 (15.9) | 1027 (15.1) |  |
| 2 to 4 | 2189 (20.7)** | 880 (22.3) | 1309 (19.8) |  |
| Missing | 27 (0.26)** | 12 (0.30) | 15 (0.23) | ꭓ ^2^ (2) =10.188 ; *p<.001* |
| Hallucinations |  |  | |  |
| 0 | 2534 (24.0)** | 952 (24.1) | 1582 (24.0) |  |
| 1 | 1776 (16.8)** | 671 (17.0) | 1105 (16.7) |  |
| 2 to 4 | 6202 (58.8)** | 2310 (58.4) | 3892 (59.0) |  |
| Missing | 42 (0.40)** | 21 (0.53) | 21 (0.32) | ꭓ ^2^(2) =0.203; *p=*.903 |
| Depressed mood |  |  | |  |
| 0 | 4486 (42.5)** | 1712 (43.3) | 2774 (42.0) |  |
| 1 | 3260 (30.9)** | 1228 (31.1) | 2032 (30.8) |  |
| 2 to 4 | 2792 (26.5)** | 1010 (25.5) | 1782 (27.0) |  |
| Missing | 16 (0.15)** | 4 (0.10) | 12 (0.18) | ꭓ ^2^(2) = 2.973; *p=.*226 |
| Other mental problems |  |  | |  |
| 0 | 3011 (28.5)** | 1178 (29.8) | 1833 (27.8) |  |
| 1 | 2185 (20.7)** | 789 (20.0) | 1396 (21.2) |  |
| 2 to 4 | 5311 (50.3)** | 1965 (49.7) | 3346 (50.7) |  |
| Missing | 47 (0.45)** | 22 (5.56) | 25 (0.38) | ꭓ ^2^(2) = 5.734; *p=.057* |
| Relationship Problems |  |  | |  |
| 0 | 3904 (37.0)** | 1472 (37.2) | 2432 (36.8) |  |
| 1 | 2733 (25.9)** | 1048 (26.5) | 1685 (25.5) |  |
| 2 to 4 | 3844 (36.4)** | 1411 (35.7) | 2433 (36.9) |  |
| Missing | 73 (0.69)** | 23 (5.82) | 50 (0.76) | ꭓ ^2^(2) 1.937; *p =.380* |
| Daily living problems |  |  | |  |
| 0 | 5188 (49.2)** | 1977 (50.0) | 3211 (48.7) |  |
| 1 | 2344 (22.2)** | 843 (21.3) | 1501 (22.7) |  |
| 2 to 4 | 2960 (28.0)** | 1114 (28.2) | 1846 (28.0) |  |
| Missing | 62 (0.59)** | 20 (0.51) | 42 (0.64) | ꭓ ^2^ (2) 3.198; *p*=.*202* |
| Living conditions |  |  | |  |
| 0 | 6086 (57.7)** | 2272 (57.5) | 3814 (57.8) |  |
| 1 | 1812 (17.2)** | 683 (17.3) | 1129 (17.1) |  |
| 2 to 4 | 2346 (22.2)** | 882 (22.3) | 1464 (22.2) |  |
| Missing | 310 (2.94)** | 117 (2.96) | 193 (2.92) | ꭓ ^2^(2)= 0.103; *p=*.950 |
| Occupational problems |  |  | |  |
| 0 | 4523 (42.9)** | 1732 (43.8) | 2791 (42.3) |  |
| 1 | 2436 (23.1)** | 921 (23.3) | 1515 (23.0) |  |
| 2 to 4 | 3320 (31.5)** | 1215 (30.7) | 2105 (31.9) |  |
| Missing | 275 (2.61)** | 86 (2.18) | 189 (2.86) | ꭓ ^2^(2) =2.392; *p=.303* |

Note. T-tests were conducted between the HoNOS scores of individuals with and without complex multimorbidity. Chi-square tests were also conducted between individuals with and without complex multimorbidity and (1) HoNOS subscales; (2) ICD-10 diagnosis and (3) Intellectual disability. *Percentage by row. **Percentages by column
